# Characterization of Plasmodium developmental transcriptomes in Anopheles gambiae midgut reveals novel regulators of malaria transmission

**DOI:** 10.1111/cmi.12363

**Published:** 2014-10-31

**Authors:** Karolina A Akinosoglou, Ellen S C Bushell, Chiamaka Valerie Ukegbu, Timm Schlegelmilch, Jee-Sun Cho, Seth Redmond, Katarzyna Sala, George K Christophides, Dina Vlachou

**Affiliations:** 1 Department of Life Sciences, Imperial College LondonLondon, UK; 2 The Cyprus InstituteNicosia, Cyprus

## Abstract

The passage through the mosquito is a major bottleneck for malaria parasite populations and a target of interventions aiming to block disease transmission. Here, we used DNA microarrays to profile the developmental transcriptomes of the rodent malaria parasite *Plasmodium berghei in vivo*, in the midgut of *Anopheles gambiae* mosquitoes, from parasite stages in the midgut blood bolus to sporulating oocysts on the basal gut wall. Data analysis identified several distinct transcriptional programmes encompassing genes putatively involved in developmental processes or in interactions with the mosquito. At least two of these programmes are associated with the ookinete development that is linked to mosquito midgut invasion and establishment of infection. Targeted disruption by homologous recombination of two of these genes resulted in mutant parasites exhibiting notable infection phenotypes. GAMER encodes a short polypeptide with granular localization in the gametocyte cytoplasm and shows a highly penetrant loss-of-function phenotype manifested as greatly reduced ookinete numbers, linked to impaired male gamete release. *HADO* encodes a putative magnesium phosphatase with distinctive cortical localization along the concave ookinete periphery. Disruption of *HADO* compromises ookinete development leading to significant reduction of oocyst numbers. Our data provide important insights into the molecular framework underpinning *Plasmodium* development in the mosquito and identifies two genes with important functions at initial stages of parasite development in the mosquito midgut.

## Introduction

Malaria remains an important public health problem despite persistent efforts for control and prevention. Disease related morbidity and mortality is the result of the parasite asexual cycle of red blood cell (RBC) invasion and intraerythrocytic replication, while disease transmission depends on a subset of parasites that escape this cycle and differentiate into gametocytes. Upon ingestion by a compatible mosquito vector, gametocytes embark on sexual reproduction and sporogonic development. Inside the mosquito midgut, male and female gametocytes rapidly produce gametes, which escape from the host cell membrane and fuse to form zygotes. Within hours, zygotes develop to motile ookinetes that escape the blood bolus-encasing peritrophic matrix and traverse the midgut epithelium. On the basal side of the epithelium, ookinetes develop to replicative oocysts where thousands of sporozoites are produced. Oocysts burst and sporozoites released into the hemocoel migrate to the salivary glands from where they are transmitted to a new host during a subsequent mosquito bite.

The gametocyte-to-ookinete-to-oocyst developmental transition is completed within the first 24 h in the mosquito midgut and is the most critical stage of the entire transmission cycle. The ingested parasite populations suffer substantial losses during this stage resulting in very few oocysts and in most cases, termination of transmission. This stage is therefore a good target of interventions aiming to control disease transmission (Aly and Matuschewski, 2005; Churcher *et al*., 2010; 2013[Bibr b7],[Bibr b8]; Griffin *et al*., 2010). However, the relatively small number of proteins that have been characterized to date with established functions in these stages does not permit comprehensive understanding of the molecular processes that control this transition stage, which could in turn inform the development of respective interventions. This is due to the rather complex regulatory mechanisms of gene expression, which hinder the identification of proteins and molecular mechanisms involved in these developmental processes.

Transcriptomic (Hayward *et al*., 2000; Mamoun *et al*., 2001; Bozdech *et al*., 2003; Le Roch *et al*., 2003; Hall *et al*., 2005; Silvestrini *et al*., 2005; Vontas *et al*., 2005; Xu *et al*., 2005; Young *et al*., 2005; Raibaud *et al*., 2006) and proteomic (Florens *et al*., 2002; Lasonder *et al*., 2002; 2008[Bibr b32],[Bibr b33]; Hall *et al*., 2005; Khan *et al*., 2005) studies, have revealed a key role of transcriptional regulation in *Plasmodium* biology. A relatively good correlation between transcript and protein temporal expression patterns has been revealed, which appears to generally apply also to late stages of the gametocyte-to-ookinete-to-oocyst developmental transition. Midgut invasion and transformation to oocyst are associated with transcripts specifically produced in the maturing zygote, e.g. the Circumsporozoite and TRAP-related protein (*CTRP*), chitinase (*CHT1*), secreted ookinete adhesive protein (*SOAP*), von Willebrand factor A domain-related protein (*WARP*) and others (Dessens *et al*., 1999; 2001; 2003[Bibr b10],[Bibr b11],[Bibr b12]; Yuda *et al*., 1999a; 2001[Bibr b57],[Bibr b59]; Tomas *et al*., 2001; Kadota *et al*., 2004; Hirai *et al*., 2006; Ishino *et al*., 2006; Kariu *et al*., 2006; Siden-Kiamos *et al*., 2006; Ecker *et al*., 2007; Bushell *et al*., 2009). However, a temporal discontinuity between transcription and translation has also been observed as maternally inherited transcripts such as those encoding the major ookinete surface proteins P25 and P28 are expressed in female gametocytes but remain translationally repressed until after fertilization (Hall *et al*., 2005; Mair *et al*., 2006; 2010[Bibr b35],[Bibr b36]). Yet the example of the male inherited sporulation factor important for transmission that is transcribed and translated in male gametocytes but has a mutant phenotype manifested during the ookinete-to-oocyst transformation highlights the great complexity of the system (Bushell *et al*., 2009).

The principal aim of this study was to generate a comprehensive catalogue of genes that are transcriptionally regulated during *Plasmodium* development in the mosquito midgut. This data could be then used to guide future systematic approaches towards a better understanding of the gametocyte-to-ookinete-to-oocyst developmental transition. For this, we carried out a comprehensive transcriptional profiling of the developmental migration of the rodent malaria parasite *Plasmodium berghei in vivo* in the Afrotropical mosquito *Anopheles gambiae*, from midgut blood bolus stages to mature sporulating oocysts, using oligonucleotide DNA microarrays. We clustered the transcriptional profiles of genes showing regulation across the various time points and, guided by the profiles of already characterized genes, generated lists of genes putatively involved in parasite developmental programmes and interactions with the vector. To assess the functional relevance of our findings, we selected for targeted gene disruption and phenotypic characterization two genes with peak transcription 24 h post-infection (hpi). This analysis showed that disruption of either of the two genes compromises the capacity of parasites to infect the mosquito midgut. Our data provide new insights into gene expression during *Plasmodium* sexual and sporogonic development in the mosquito and identifies novel potential targets of malaria transmission blocking interventions.

## Results

### Transcriptional profiling of *P. berghei* in the *A. gambiae* midgut

We carried out three independent biological replicate infections of *A. gambiae* with the 259c12 line of *P. berghei* that constitutively expresses GFP (Franke-Fayard *et al*., 2004). For each replicate, RNA was prepared from 30–40 mosquito guts at six discrete time points post-infection; mixed asexual and sexual stages in the blood bolus (1 h), ookinete midgut invasion (24 h), early-stage oocysts (48 h), mid-stage oocysts (5 days), sporulating oocysts (10 days) and mature oocysts (13 days). Blood boluses were removed from 48 h guts to eliminate remnants of blood stage parasites.

To test the suitability of RNA samples, we performed three quality control assays. (i) We assessed the intensity of infection by examining the presence of oocysts in mosquito midguts at day 10. The average oocyst counts were 25.9, 32.1 and 40.7 in the three replicate infections respectively. (ii) As parasite developmental dynamics naturally fluctuate between infections, we carried out a quantitative real-time PCR (qRT-PCR) analysis of the *GFP* transcript levels in each of the time points and replicate infections. In each replicate, the GFP expression in each time point was referenced to the average GFP expression across all the time points. In all the replicates, the infection dynamics inferred from the *GFP* levels indeed resembled the expected parasite population dynamics in the mosquito midgut (Supporting Information Fig. S1A). (iii) We examined the expression of three stage-specific genes by qRT-PCR, including the gametocyte-specific *P28* gene, the zygote- and ookinete-specific *CTRP* gene and the sporozoite-specific circumsporozoite protein (*CSP*) gene. All the genes exhibited expected expression profiles (Supporting Information Fig. S1B–D).

We used the RNA samples in hybridizations of oligonucleotide DNA microarrays (Mair *et al*., 2006; Bushell *et al*., 2009). Our design involved hybridization of each of the RNA samples against a standard reference RNA that was a mixture of the RNA samples of each replicate infection, pooled according to the *GFP* expression as described in the ‘Experimental procedures’. This design was chosen to ensure that all transcripts were represented in the reference sample.

Mapping of the oligonucleotide probes revealed that the microarrays encompassed 4284 of the 5164 genes predicted in the *P. berghei* genome according to the PlasmoDB 11.1 May 2014 release (i.e. 83% coverage). Of these, 3454 genes were identical to those included the original array design and were represented by the same probes, while 830 resulted from merging or splitting of genes between genebuilds and thus were represented by a different combination of probes. To preclude differential handling, the latter gene group was not considered further.

**Figure 1:**
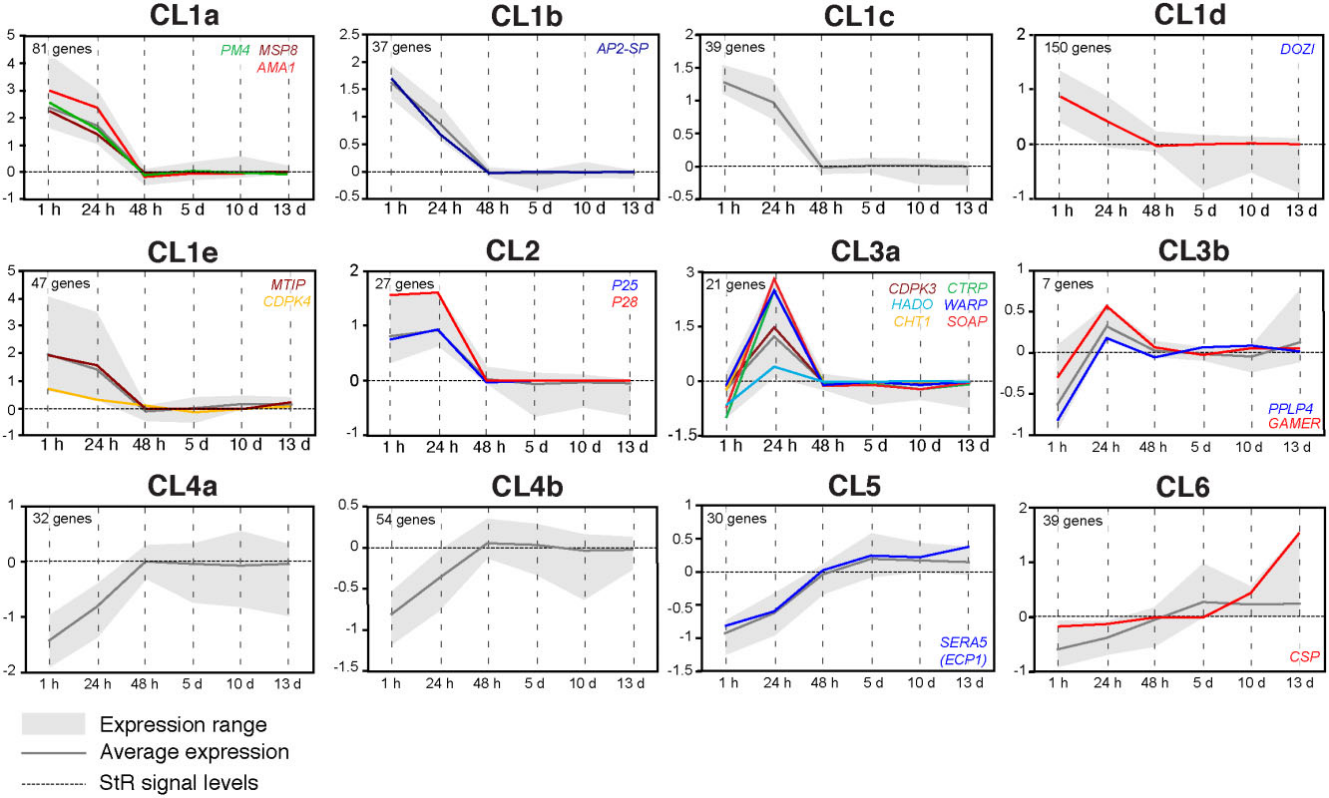
*Plasmodium berghei* developmental co-expression clusters in the *A. gambiae* midgut. Transcriptional co-expression gene clusters that together depict the *P. berghei* developmental expression in the mosquito midgut from early blood bolus to late oocyst stages. In generating these clusters, genes exhibiting significant (*P* < 0.05) differential regulation between any two time points, assessed with one-way ANOVA, and at least 0.8-fold difference in a log_2_ scale between their minimum and maximum expression after correction with the Benjamini-Hochberg hypergeometric test were grouped using a combination of SOM and K-means clustering. The *Y*-axis scale shows expression relative to the standard reference sample in log_2_-transformed values; horizontal dashed lines indicate the signal levels of the standard reference. Black solid lines indicate the average expression of all the genes in each cluster and grey areas indicate their range of expression. Genes with known developmental expression profiles are shown with coloured solid lines. The number of genes in each cluster is indicated at the top left corner of each graph.

Data normalization and one-way analysis of variance (ANOVA) statistical tests on gene expression data following correction with the Benjamini-Hochberg hypergeometric test (see ‘Experimental procedures’) revealed that 1639 of 3454 genes exhibited statistically significant differential regulation between at least two of the time points (*P* ≤ 0.05; Supporting Information Table S1). Of these, 564 genes showed at least 0.8 log_2_-transformed fold difference between their minimum and maximum expression (Supporting Information Table S2). Clustering of the expression profiles of these 564 genes using a combination of self-organizing maps (SOM) and K-means clustering revealed the presence of a total of 12 co-expression clusters: six main clusters, of which four are divided into sub-clusters. These clusters together depicted the *P. berghei* developmental transcriptome in the mosquito midgut (Fig. 1 and Supporting Information Table S2). In support of our approach, genes with known developmental expression profiles were mapped in expected clusters and used to guide the annotation of the expression map.

### Characterization of developmental transcription profiles

Genes exhibiting enriched expression in blood bolus stages (mixed asexual and sexual stages) are grouped into two highly populated co-expression clusters (CL1 and 2), together encompassing 381 genes (Fig. 1 and Supporting Information Table S2). CL1 is divided into five sub-clusters (CL1a-e), differentiated between them mainly based on the levels of differential regulation, together comprising 354 genes with transcript levels decreasing after 1 hpi. *MSP8* (Black *et al*., 2001) and *AMA1* (Narum and Thomas, 1994), the transcription factor *AP2-SP* (Yuda *et al*., 2010), the RNA helicase *DOZI* (Mair *et al*., 2010), *MTIP* (Bergman *et al*., 2003) and the calcium-dependent protein kinase *CDPK4* (Billker *et al*., 2004) belong to CL1. The master regulator of male-specific events during gametogenesis CDPK4 (as well as MTIP) is found in CL1e, which is characterized by an additional slightly increased expression during sporogony. Indeed, it was previously hypothesized that CDPK4 also has an unknown function during sporogony (Billker *et al*., 2004). Quantitative real-time PCR analysis confirmed the expression of *CDPK4* at 10 dpi (Supporting Information Fig. S2). CL2 contains 27 genes that are highly expressed in both 1 and 24 hpi, including the major zygote and ookinete surface proteins *P25* and *P28* (Paton *et al*., 1993).

CL3 is composed of two sub-clusters. CL3a is a tight cluster comprised of 21 genes exhibiting a sharp peak in transcript abundance at 24 hpi, suggesting enriched or specific expression in ookinetes (Fig. 1 and Supporting Information Table S2). Six of these genes have been previously implicated in mosquito midgut invasion, including *CTRP* (Dessens *et al*., 1999), *SOAP* (Dessens *et al*., 2003), *CHT1* (Dessens *et al*., 2001), *CDPK3* (Ishino *et al*., 2006), *WARP* (Yuda *et al*., 2001) and *PSOP2* (Ecker *et al*., 2008). Of the remaining 15 genes, five encode proteins bearing domains implicated in metabolic and housekeeping functions and 10 are of unknown function. Putative AP2-O transcription factor binding sites (Yuda *et al*., 2009) are found within a 1 kb region upstream of the open reading frames (ORFs) for 17 of these genes (Supporting Information Table S2), indicative of the ookinete specificity of this cluster.

CL3b is a small cluster that includes six previously uncharacterized genes showing increased expression at both 24 hpi and maturing oocysts. This cluster involves the *Plasmodium* perforin-like protein, PPLP4 (Ecker *et al*., 2008). Quantitative real-time PCR analysis confirmed expression of *PPLP4* in oocysts (Supporting Information Fig. S2).

Very little is known about the type of gene products required in the developing oocyst. Our analysis detected a large shift in gene expression as the ookinete transitions to oocyst. Genes exhibiting enriched expression in oocyst stages are grouped into three main clusters (CL4-6 in Fig. 1 and Supporting Information Table S2) together encompassing 155 genes.

CL4 is a highly populated cluster of 86 genes that are up-regulated as parasites develop from blood bolus stages to ookinete and to oocyst and retain their expression levels throughout oocyst development. CL4 is further divided into two sub-clusters, CL4a and CL4b, respectively, based on the level of differential regulation between the first three time points.

CL5 includes 30 genes with increasing expression throughout oocyst development. It includes the serine repeated antigen 5 (*SERA5*) gene that is essential for sporozoite egress from the oocyst (Aly and Matuschewski, 2005).

Finally, CL6 involves 39 genes exhibiting expression similar to those in CL5 but characterized by increased expression at 13 dpi. It involves *CSP* that is known to be expressed in sporozoites and be essential for their development (Ménard *et al*., 1997).

In addition to using genes with known expression profiles to validate the profiling, we used qRT-PCR for a number of genes to test the power of our approach in discovering novel genes with temporally regulated expression (Supporting Information Fig. S2). We carried out three new experimental replicate infections of *A. gambiae* mosquitoes with the 259c12 *P. berghei* line, and prepared RNA samples from the six time points using a protocol identical to that we used for the DNA microarray analysis. We examined the expression of 12 genes including seven novel genes, two known genes with newly detected expression in the oocyst stage, *CDPK4* and *PPLP4*, and the *P28*, *CTRP* and *CSP*; the latter three served as stage-specific controls (Supporting Information Fig. S2). The *GFP* transgene served as an internal reference for constitutive expression. An overall great consistency was observed between the DNA microarray and qRT-PCR expression profiles for all the genes tested. Minor relative quantitative differences are thought to be due to the different normalization methods and the different sets of probes/primes used in each approach.

### Characterization of *GAMER* and *HADO*

We focused downstream analysis on genes putatively involved in ookinete development and transformation to oocyst, i.e. genes showing peak expression 24 hpi and included in CL3a and CL3b. Genes exhibiting *P25* and *P28*-like profiles, i.e. transcriptionally peak in ookinetes but are abundantly expressed also in blood bolus stages, and included in CL2, were not considered in the present study.

We prioritized for genetic characterization genes of which (i) orthologues exist in *P. falciparum* and *P. vivax*, (ii) gene models could indicate feasibility of targeted disruption by homologous recombination, including high-quality sequences for successful design of disruption vectors and sufficient distance from neighboring genes to preclude non-specific gene knockout and (iii) predicted protein domains did not hint at housekeeping functions. Here, we present the genetic and phenotypic characterization of two genes: PBANKA_122540 and PBANKA_060390, called *GAMER* and *HADO*, respectively, for reasons explained below.

**Figure 2:**
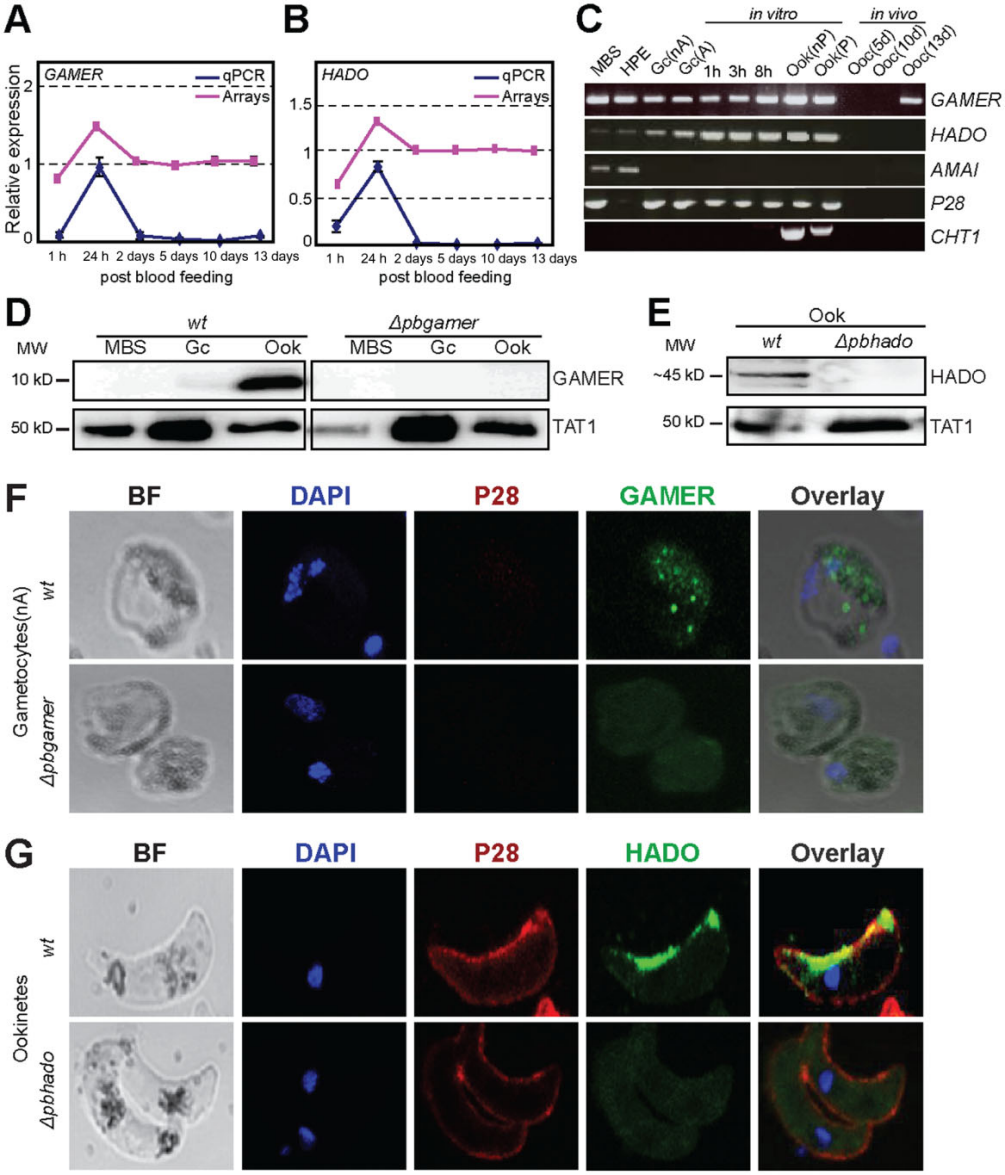
Expression and subcellular localization of *P. berghei* *GAMER* and *HADO*.A, B. Transcriptional expression profiles of *GAMER* and *HADO*, respectively, throughout *Plasmodium* development in the mosquito midgut. The DNA microarray (purple line) and qRT-PCR (blue line) profiles are shown. The vertical axis indicates the relative expression. Quantitative real-time PCR data are the mean of three biological replicates (fully independent infections) each derived from two technical replicates. Data were normalized to the level of constitutively expressed *GFP* transcripts. Error bars indicate standard error of the mean.C. RT-PCR analysis of *GAMER* and *HADO* in asexual blood stages using the non-gametocyte producing strain (HPE); mixed blood stages (MBS); activated (A) and non-activated (nA) gametocytes (Gc); 1 h, 3 h and 8 h zygotes; and non-purified (nP) and purified (P) *in vitro* produced ookinetes (Ook). The analysis was complemented with *in vivo* 5, 10 and 13 day (d) oocysts. *P28*, *AMA1* and *CHT1* served as stage-specific and loading controls.D. Western blot analysis of *wt* parasites using an anti-GAMER peptide antibody (a-GAMER). Knockout *Δpbgamer* parasites were used to control for non-specific signal. Anti-TAT1 (a-TAT1) antibody was used as internal control.E. Western blot analysis of *wt* ookinetes using an anti-HADO peptide antibody. *Δpbhado* ookinete protein extracts were used to control for non-specific signal.F, G. Immunofluorescence assays of purified *P. berghei* ANKA 2.34 *wt* mature, non-activated (nA) gametocytes (upper panels) labelled for P28 (red), DAPI (blue) and GAMER (green; F) or HADO (green; G) respectively. Bright field (BF) visualization is also shown. IFA of *Δpbgamer* and *Δpbhado* gametocytes, respectively, served as a negative control. Images were taken from confocal sections of fixed parasites.

The expression profiles of *GAMER* and *HADO* were confirmed by qRT-PCR (Fig. 2A and B). We also examined the expression patterns of the two genes in early stages of sexual development using RT-PCR on purified *in vivo* and *in vitro* cultured parasite stage-specific populations (Fig. 2C). The combination of these assays revealed that *GAMER* and *HADO* are expressed in gametocytes and further up-regulated in the zygote and ookinete. *HADO* shows paucity of expression throughout oocyst development whereas *GAMER* is re-expressed in a 13 day oocyst, presumably in sporozoites. Literature searches revealed that *GAMER* transcripts are down-regulated in gametocytes lacking CITH (CAR-I and fly Trailer hitch), the interacting partner of DOZI (Mair *et al*., 2010), while *HADO* transcripts are moderately enriched in gametocytes lacking DOZI (Mair *et al*., 2006). DOZI and CITH are repressors of maternally supplied mRNA important for ookinete development (Mair *et al*., 2010). Interestingly, putative *P. berghei* ookinete transcription factor AP2-O binding sites are found within 200–400 bp upstream of the predicted *GAMER* and *HADO* ORFs (Supporting Information Table S2).

*GAMER* encodes a small 96 amino acid protein (10.7 kD) lacking recognizable domains, as revealed by both manual annotation and three-dimensional (3D) homology modelling. The deduced protein is conserved among plasmodia with 91%, 86%, 71%, 70% and 65% identity to *P. yoelii* PYYM_1228100, *P. chabaudi* PCHAS_122600, *P. knowlesi* PKH_011340, *P. vivax* PVX_093500 and *P. falciparum* PF3D7_1205200 respectively (Supporting Information Fig. S3). We produced rabbit polyclonal antibodies against the C-terminal peptide SEKAKELLRERGYVV (EP092419; 82–96 aa) of *P. berghei* GAMER. Western blot analysis of mixed blood stages, gametocytes and ookinetes confirmed that GAMER is a 10 kDa polypeptide specifically produced in ookinetes (Fig. 2D). However, immunofluorescence assays (IFA) on *in vitro* purified (non-activated) gametocytes using GAMER antibodies revealed protein presence in the gametocyte cytoplasm, where it localizes to discrete foci possibly of vesicular nature (Fig. 2F). IFA on purified ookinetes showed a diffused signal in the ookinete cytoplasm (data not shown).

*HADO* encodes a 379 amino acid protein (44.7 kD) that is also well conserved among plasmodia with 95%, 83%, 78%, 76% and 70% identity to PY05386, PF3D7_1205200, PCHAS_060570, PVX_084290 and PKH_130370 respectively (Supporting Information Fig. S4). Manual annotation and homology modelling analysis revealed that *HADO* encodes a haloacid dehalogenase (HAD) domain protein with structural similarity to the human magnesium-dependent phosphatase Mdp-1 (Supporting Information Fig. S5; Seifried *et al*., 2013). The name HADO was derived from HAD domain ookinete protein. The FDYDDTI motif of the HAD domain is conserved in all *Plasmodium* orthologues and the conserved 3D models suggest a putative DxDxT phosphatase with a very high probability (Supporting Information Figs S4 and S5). Western blot analysis using a rabbit polyclonal antibody raised against the C-terminal peptide VFPINFKDRNSIKNL (240–254 aa) of HADO identified a ∼ 45 kD protein expressed in ookinetes (Fig. 2E), while preliminary data indicated low expression in purified gametocytes that was below threshold expression in mixed blood stages. IFA of *in vitro* purified ookinetes using the *P. berghei* HADO antibody revealed a distinctive cortical localization along the concave ookinete periphery (Fig. 2G).

### Generation and phenotypic analysis of *Δpbgamer* and *Δpbhado* mutant parasites

*Plasmodium berghei* mutants were generated by replacing the entire *GAMER* and large parts of the *HADO*-coding regions with modified *Toxoplasma gondii* pyrimethamine resistance cassettes in the Pbc507 GFP-expressing parasite reference line (Janse *et al*., 2006a; Supporting Information Fig. S6A and B). Integration of the disruption cassettes and disruption of each of the two genes in clonal parasite lines was verified by PCR (Supporting Information Fig. S6C) and Southern analysis of separated chromosomes and of digested genomic DNA (Supporting Information Fig. S6D). At least two clonal mutant lines per gene were generated from respective independent transfections and analysed as described below.

**Figure 3:**
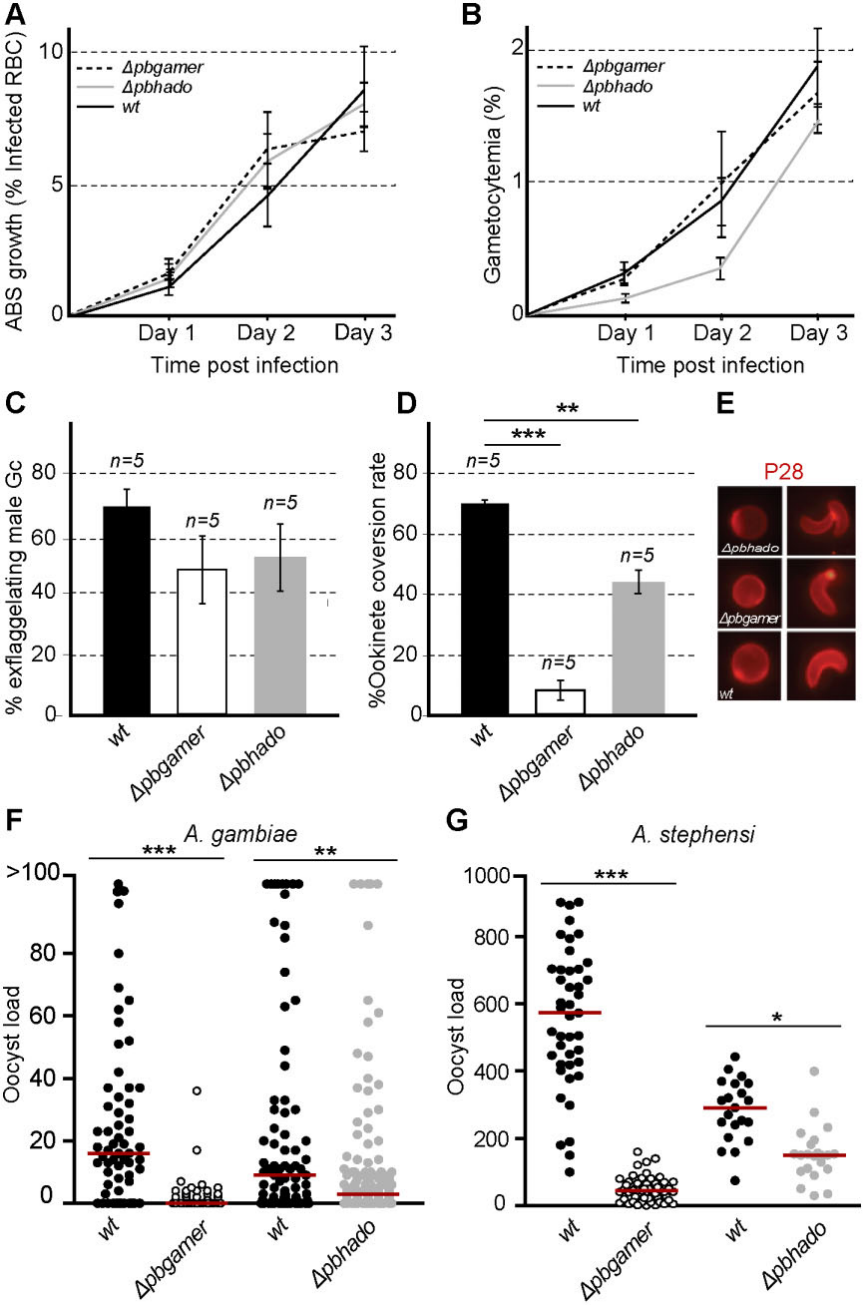
Phenotypic analysis of *Δpbgamer* and *Δpbhado* mutant parasites.A. Asexual blood stage growth corresponding to the percentage of infected RBCs.B. Gametocytemia, indicating the percentage of infected RBCs that are at the gametocyte stage.C. Exflagellation assays, showing the percentage of male gametocytes that form exflagellation centres.D. Gametocyte-to-ookinete conversion ratio. Both the exflagellation and the gametocyte-to-ookinete conversion assays were repeated five times and statistical significance determined with a two tailed, unpaired Student's *t*-test.E. Distribution of P28.F, G. Oocyst loads at day 10 pi in the midguts of *A. gambiae* and *A. stephensi* mosquitoes respectively. Stars indicate statistical significance determined with Mann–Whitney *U*-test. The median is shown with a red line. ***P < 0.0001; **P < 0.001, *P < 0.05. *n*, number of independent biological replicates. Error bars indicate standard error of mean.

Phenotypic analysis revealed that both mutants exhibited normal development of asexual blood stages and mature gametocytes, while activation of male gametogenesis, as measured by the formation of exflagellation centres, was also normal in both mutants (Fig. 3A–C). However, macrogamete to ookinete conversion was markedly reduced to 18% (*P* < 0.0001) and 42% (*P* < 0.01) for both *Δpbgamer* and *Δpbhado*, respectively, compared with 71.5% in *wt* parasites (Fig. 3D). Both, *Δpbgamer* and *Δpbhado* macrogametes and ookinetes, albeit reduced in number, were morphologically normal and exhibited an expected surface distribution of P28 (Fig. 3E).

We assessed the ability of mutant parasites to develop to oocysts in both *A. gambiae* and *A. stephensi* mosquitoes that were fed on mice infected with each of these mutant or control *wt* parasites. Mice with a parasitaemia of 6–7% and gametocytaemia 1–2% were used for mosquito infections. The numbers of oocysts were determined 10 dpi. The results showed that, compared with *wt* controls at day 10 pi, oocyst numbers were significantly reduced in both *Δpbgamer* and *Δpbhado* mutants, reflecting prior gamete-to-ookinete developmental defects (Fig. 3F and G and Supporting Information Table S3). The numbers of salivary gland sporozoites of the two mutants were also significantly reduced compared with *wt* controls respectively (Supporting Information Table S4). Mutant parasites that developed to normal oocysts and infect the salivary glands were able to be transmitted to the vertebrate host by both *A. gambiae* and *A. stephensi* mosquitoes as revealed by bite-back experiments at days 18 and 21 dpi using susceptible BL6/C56 recipient mice (Supporting Information Table S4).

### GAMER is essential for gamete release

**Figure 4:**
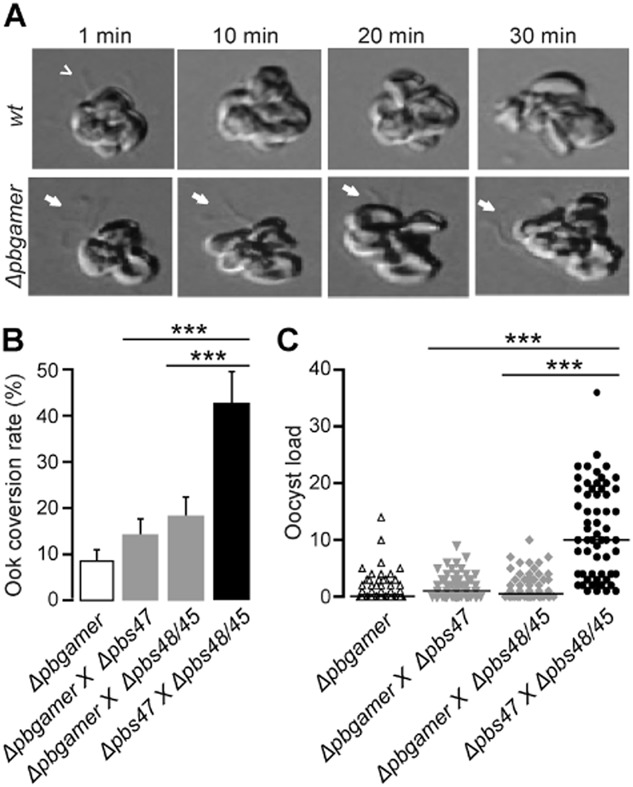
*Δpbgamer* exflagellation and genetic complementation assays.A. *Wt* and *Δpbgamer* exflagellation centres at 1, 10, 20 and 30 min post-male gametocyte activation. Images were obtained by time-lapse microscopy at 43× magnification. Incomplete detachment of male gametes (white arrows) is observed for *Δpbgamer* as opposed to complete release of male gametes (white arrowhead) in *wt* parasites at 30 min post-male gametocyte activation.B. Gametocyte-to-ookinete conversion ratios in genetic crosses of *Δpbgamer* with either male-, *Δpbs48/45*, or female-deficient lines, *Δpbs47*, showing no phenotype rescue. Genetic crosses between *Δpbs48/45* and *Δpbs47* were used as a positive control. The arithmetic mean and standard error of the mean (SEM) are shown. Three biological experiments were performed for each genetic cross group and *P*-values were calculated with the Student's *t*-test.C. Oocyst distribution in the midguts of *A. gambiae* mosquitoes at day 9 pi, following genetic crossing *of* *Δpbgamer* with either *Δpbs48/45* or *Δpbs47*-deficient lines. Three biological experiments were performed for each of the genetic crosses group and statistical significance was determined with the Mann–Whitney *U*-test. The median is shown with a black line. Stars indicate statistical significance; ***P < 0.0001.

The detection of GAMER in gametocytes prompted us to investigate whether the effect of gene disruption in ookinete development is due to defects prior to or after fertilization by monitoring the exflagellation event at real time using time-lapse microscopy. The results showed that activated *Δpbgamer* male gametocytes produce morphologically normal and motile microgametes; however, the vast majority of them was unable to detach from the residual body of the gametocytes and remained attached to the exflagellation centres even 30 min post-activation (Fig. 4A). This phenotype is consistent with a putative function of GAMER in male gametocytes. The name GAMER is therefore derived from GAMEte Release.

We investigated further the *Δpbgamer* male gamete release phenotype through genetic crosses with *P. berghei* mutants producing fertile gametes of one sex but not the other. Indeed, genetic crosses with *Δpbs48/45*, which contribute fertile female but not male gametes, could not rescue the mutant phenotype (Fig. 4B). Surprisingly, similar results were obtained when *Δpbgamer* was crossed to *Δpbs47*, which contribute fertile male but not female gametes, i.e. the mutant phenotype could not be rescued. These data indicated that *Δpbgamer* macrogametes are also defective, despite the apparently normal distribution of P28. It remains to be investigated whether GAMER is indeed involved in both male and female gametocyte release from the erythrocyte membrane. Equivalent results were obtained *in vivo*, in *A. gambiae* infected with *Δpbgamer* crossed with either *Δpbs47* or *Δpbs48/*45 respectively. Neither the male nor the female alleles alone could restore the oocyst numbers, confirming that the existence of functional forms of both parental alleles is essential for mosquito infection (Fig. 4C and Supporting Information Table S6).

## Discussion

Malaria remains one of the most devastating diseases causing high morbidity and mortality despite persistent efforts for elimination. The inadequacy of current preventive measures can be largely attributed to the complex developmental life cycle of the malaria parasite in its human host and mosquito vector. Elucidation of the molecular mechanisms underlying parasite development and the interactions with its host and vector can provide fundamental insights into *Plasmodium* biology, which could eventually help in the design of novel disease elimination strategies.

Despite the wealth of transcriptome and proteome data on the asexual *Plasmodium* life cycle in the vertebrate host blood, mechanisms and molecules involved in vector stages of the life cycle remain poorly understood. Here, we report the profiling of the developmental transcriptomes of the rodent malaria parasite *P. berghei* in the midgut of the major African vector, *A. gambiae*, from blood bolus stages to mature oocysts in order to gain molecular insights into this critical phase of malaria transmission. This phase encompasses three major developmental processes: sexual reproduction (including gametogenesis and fertilization), meiotic development of the zygote/ookinete and vegetative growth of the oocyst via endomitotic replication. These processes are separated from each other by only few hours, while the transition between the latter two processes requires traversal of the mosquito midgut, a step that corresponds to the most critical population bottleneck of the entire parasite life cycle; more often than not, transmission is terminated at this very stage.

Our data provide new insights into the transcriptional programmes underpinning these developmental processes with a large number of genes found to be expressed between the various time points. These genes are grouped into distinct transcriptional programmes according to their expression profile, some of which operate during the various developmental processes, while others are involved in transition stages.

While epigenetic (Lopez-Rubio *et al*., 2009) and post-transcriptional regulation (Mair *et al*., 2006; 2010[Bibr b35],[Bibr b36]) have emerged as significant regulatory mechanisms of gene expression, the AP2 protein family of transcriptional regulators is thought to be the fundamental machinery guiding gene expression throughout *Plasmodium* life cycle (Painter *et al*., 2011). AP2 proteins are shown to regulate the asexual to sexual life cycle switch (Kafsack *et al*., 2014; Sinha *et al*., 2014), the ookinete and sporozoite development in the vector (Yuda *et al*., 2009; 2010[Bibr b60],[Bibr b61]) and the sporozoite development in the host liver (Iwanaga *et al*., 2012). The AP2-O (Yuda *et al*., 2009) appears to be particularly relevant in understanding our data. It is specifically transcribed in the female *P. berghei* gametocyte, and its transcript that is translationally repressed in a DOZI-dependent manner becomes available for translation in the zygote/ookinete (Yuda *et al*., 2009). There, it drives the transcription of genes largely involved in midgut invasion including *CTRP*, *SOAP*, *CHT1*, *WARP*, *P25*, *P28*, *CDPK3* and *PSOPs* (Yuda *et al*., 2009).

A key objective of this work has been the molecular dissection of midgut invasion that coincides with the ookinete-to-oocyst developmental transition. Indeed, we detected a cluster of 21 genes (CL3) exhibiting a sharp peak in transcript abundance at 24 hpi, suggesting that these genes are substantially enriched or specifically expressed in midgut invading ookinetes, including *CTRP*, *SOAP*, *CHT1*, *WARP*, *CDPK3* and *PSOP2*. Putative AP2-O transcription factor binding sites (Yuda *et al*., 2009) are present in the upstream region of 21 of these 28 genes indicative of the ookinete specificity of this cluster. The phenotypic and functional characterization of *HADO* confirmed the functional identity of this cluster by revealing a new regulator of ookinete development. Therefore, CL3 appears to represent a classical example of AP2-O-regulated cluster, in which gene transcription is tightly linked to gene function.

*HADO* encodes a HAD domain phosphatase with a DxDxT catalytic motif, structurally similar to human Mdp-1 (Seifried *et al*., 2013). Proteins with this domain are known to be involved in the regulation of the actin dynamics through cofilin dephosphorylation. The distinctive cortical localization of HADO along the concave side of ookinetes supports the hypothesis that the protein may function in regulating the cellular actin dynamics, maybe towards carrying out or transitioning between specific movements types that take place during midgut invasion (Vlachou *et al*., 2004; Kan *et al*., 2014). Along these lines, it remains to be investigated whether *HADO* mutant parasites exhibit specific defects in ookinete motile behaviour. Nevertheless, HAD domain magnesium-dependent phosphatases are also known to play roles in lipid-associated cell signalling and metabolism, including Mdp-1, which functions to free proteins from glycation products. An alternative hypothesis for the function of HADO derives from the characterization of a DxDxT phosphatase in *Trypanosoma brucei*, which regulates differentiation in the tsetse fly via a glycosomal signalling pathway, activating major changes in parasite physiology that permit vector colonization (Szöőr *et al*., 2010).

The second gene we characterized in this study, *GAMER*, is found in CL3b that includes genes sharing transcriptional enrichment in ookinetes and midgut sporozoites. The function of GAMER in male gamete egress from the host erythrocyte together with our discovery that neither the male nor the female *GAMER* alleles can restore the mutant phenotype makes it likely that the protein is also involved in female gametocyte egress from host erythrocytes. Indeed, it has been suggested that both male and female gametocyte egress is governed by common mechanisms implicating PbMDV/PEG3 (Ponzi *et al*., 2009). This protein is involved in destabilization of the parasitophorous vacuole membrane, prior to erythrocyte membrane lysis in a yet unknown mechanism. Therefore, similarly to *GAMER*, both the male and the female *PbMDV/PEG3* alleles are required.

Parasite egress has been linked to gametocyte organelles (Wirth and Pradel, 2012). Both PbMDV/PEG3 and the gametocyte-specific protein Pfg377, which is also involved in egress of *P. falciparum* female gametes, are localized in osmiophilic bodies. Moreover, the perforin-like protein PPLP2 that also mediates gamete egress in both *P. falciparum* and *P. berghei* localizes in *P. falciparum* gametocyte vesicles other than Pfg377-positive osmiophilic bodies (Deligianni *et al*., 2013; Wirth *et al*., 2014). These data highlight the important and likely synergistic function of vesicles in gamete egress and are consistent with the putative function and vesicular localization of GAMER.

In sharp contrast to the small number and fine temporal regulation of genes in CL3 is the large number of genes expressed throughout the first 24 h in the mosquito midgut, which make CL1 and CL2, the expression of which progressively declines. This study was not designed to distinguish between sexual stage expression and asexual stage contamination in the blood bolus; however, previous *in vitro* studies in *P. falciparum* have shown that orthologues of the vast majority of genes in these clusters are already expressed in gametocytes and/or gametes (Florens *et al*., 2002; Lasonder *et al*., 2002), while some are also expressed in asexual stages (Le Roch *et al*., 2003). Together these data support the hypothesis that ookinete development is supported by at least two distinct transcriptional programmes: constitutive expression throughout sexual and meiotic development (CL1 and CL2) and transient expression in ookinetes (CL3). The second programme appears to be regulated mainly by AP2-O and includes genes linked to midgut invasion, some with confirmed localization in micronemes. Transcription of these genes appears to be initiated 8–10 h post-fertilization, and their expression remains active until the transformation to oocyst (Yuda *et al*., 1999b; 2001[Bibr b58],[Bibr b59]).

Commitment to sexual differentiation in the vertebrate host is mediated by significant changes in the transcriptional repertoire (Hall *et al*., 2005; Young *et al*., 2005), while asexual replication through schizogony is characterized by cyclical expression of distinct subsets of genes at each different stage (Mamoun *et al*., 2001; Bozdech *et al*., 2003). Here we show that the same is true for parasite development in the mosquito vector. The transition from the sexual, meiotic development in the mosquito midgut lumen to the asexual sporogonic development in the mosquito hemolymph is associated with a large shift in parasite transcriptional repertoire. At the same time, transcriptional activity in the oocyst seem to follow three main patterns, which in our assays appear to overlap significantly, perhaps because of asynchronous development of oocysts between, as well as within, mosquitoes. These patterns include the onset of oocyst development (CL4), maturation (CL5) and sporulation (CL6), and they may represent distinct transcriptional programmes.

The first of these programmes is very transient and presumably the most important with respect to perpetuation or termination of transmission. At this stage, the motile ookinete completes its meiotic division and transitions to a sessile stage, preparing for sporogony. The second programme concerns rapid vegetative growth through endomitotic replication and mirrors erythrocytic schizogony. It involves a large number of largely uncharacterized genes, which are predicted to be associated with general housekeeping processes such as transcription, translation, protein processing, signalling, cell cycle regulation, lipid metabolism, organelle transport and vesicle trafficking. This programme may be separated into smaller sub-programmes corresponding to the different stages of sporogony. The third programme includes genes expressed during late oocyst development, presumably involved in sporulation or sporozoite development and migration. This involves a cluster of 39 genes that alone cannot possibly support the function of sporozoites, including cell motility and other housekeeping processes. Therefore, we hypothesize that, like ookinetes, sporozoites are supported by at least two main programmes, a generic one that operates throughout oocyst development and a specific one that specifically operates during late sporogonic stages. Functional characterization of genes in these programmes will provide important insights into this late developmental phase in the vector, which is of high importance for transmission of the disease.

## Experimental procedures

### Ethics statement

This study was carried out in strict accordance with the United Kingdom Animals (Scientific Procedures) Act 1986. The protocols for maintenance of mosquitoes by blood feeding and for infection of mosquitoes with *P. berghei* by blood feeding on parasite-infected mice were approved and carried out under the UK Home Office License PLL70/7185 awarded in 2010. The procedures are of mild to moderate severity and the numbers of animals used are minimized by incorporation of the most economical protocols. Opportunities for reduction, refinement and replacement of animal experiments are constantly monitored, and new protocols are implemented following approval by the Imperial College London Ethical Review Committee.

### Parasite cultivation and mosquito infections

*Plasmodium berghei* strains ANKA 2.34 and non-gametocyte producer HPE cy1m50 cl1, the GFP-expressing reference lines, 259c12 (Franke-Fayard *et al*., 2004) and *507* (Janse *et al*., 2006a) and the *Δpbs48/45* (RMgm-346) and *Δpbs47* (van Dijk *et al*., 2010) mutant lines (kindly provided by Chris Janse) were used in this study. Parasite handling and purification were performed as described (Janse *et al*., 2006b). *A. gambiae* Yaoundé and *A. stephensi* SD500 were reared and infected with *P. berghei* by direct feeding on infected mice with parasitaemia of 6–7% and gametocytaemia 1–2%, respectively, using standard protocols (Sinden, 1997).

### DNA microarrays

The Agilent oligonucleotide *P. berghei* microarray platform is described previously (Bushell *et al*., 2009). Remapping of oligonucleotide probes on the latest *Plasmodium* genome assembly and annotation (PlasmoDB 11.1 May 2014) revealed coverage of 4284 of the 5164 predicted genes. Of these, 3454 genes were the same as in the original microarray design and represented by the same probes, while 830 genes resulted from merging or splitting of genes between the various genebuilds and were represented by a different combination of probes compared with the original probe combinations. The latter gene group was not considered further to preclude errors due to gene annotation problems.

For hybridizations, total RNA was extracted from 30–40 *A. gambiae* midguts infected with the 259c12 parasite line at 1 h, 24 h, 48 h and 5, 10 and 13 days (d) post-infection, from three replicate infections. The midguts were dissected in ice-cold phosphate-buffered saline (PBS) under a dissecting scope and immediately immersed in Trizol® reagent (Invitrogen). They were mechanically homogenized and total RNA was isolated according to the manufacturer's instructions under RNAse-free conditions. Total RNA was quantified using a NanoDrop® ND-1000 Spectrophotometer (Thermo Scientific) and stored at − 80°C.

A standard reference was generated for each replicate infection by pooling time point samples based on the GFP expression. Specifically, to calculate the proportional amount of total RNA of each time point to be contributed to the standard reference and ensure a more-or-less uniform representation of all the time points, the average GFP expression across time points was divided by the product of the GFP expression in that time point and the sum of GFP expression values in all the time points.

The standard reference was processed in parallel to and under the same conditions as the labelled RNA from each of the time points. Briefly, from each time point sample and the standard reference, 2 μg were used for generation and labelling of cRNA using the Agilent Low RNA Input Fluoresence Amplification Kit Protocol according to manufacturer's instructions. Two micrograms of Cy3 and Cy5 labelled cRNAs, from a time point sample and the standard reference, respectively, were mixed and competitively hybridized on the Agilent microarrays using the *in situ* Hybridisation Kit Plus according to the manufacturer's instructions.

The hybridized microarrays were scanned using the Gene-Pix 4000B scanner (Axon Instruments). Grid alignment, registering spot signal intensity, estimation of local backgrounds and manual inspection of spot quality were carried out using Gene-Pix Pro 6.1. Normalization of data was achieved using the linear regression method (Lowess) in GeneSpring GX 12.6 (Agilent Technologies).

Normalization of data was performed using the locally weighted linear regression method (Lowess) in GeneSpring GX 12.6 (Agilent Technologies). Significant transcriptional differences across infection stages were calculated using a one-way ANOVA with a *P*-value cut-off of 0.05, following correction with the Benjamini-Hochberg hypergeometric test. Co-expression analysis was carried out for genes exhibiting at least 0.8-fold change in the log_2_ scale between their minimum and maximum expression values using a combination of SOM and K-means analysis. Co-expression clusters were visualized using the Cluster software version 2.11 and Java Tree View software version 1.1.6 (Eisen *et al*., 1998).

### Transcriptional profiling using qRT-PCR or RT-PCR

Total RNA was isolated from erythrocytic and sexual parasite stages and from infected mosquito midguts using the Trizol® reagent (Invitrogen). Gene-specific primers (Supporting Information Table S5) were designed using Primer3 (v. 0.4.0). Quantitative real-time PCR was carried out using SYBR-Green and the ABI Prism 7700 Sequence Detector (Applied Biosystems). *GAMER* and *HADO* transcript levels were normalized against transgenic *GFP* transcripts that provided an internal reference for the fluctuation in parasite numbers during development.

### Generation of transgenic parasites

Targeted disruption of *GAMER* and *HADO* was carried out by double homologous recombination in the *P. berghei* ANKA 2.34 or c507 genetic backgrounds as described (Janse *et al*., 2006b). Briefly, 500–1000 bp regions upstream and downstream of each target gene were amplified from *P. berghei* genomic DNA using oligonucleotide primers carrying restriction enzyme sites: ApaI/HindIII for upstream and EcorI/BamHI for downstream regions (Supporting Information Table S5). PCR products were purified using a PCR purification kit (Qiagen) and cloned into the pBS-TgDHFR vector that encompasses polylinker sites flanking the modified *Toxoplasma gondii* dihydrofolate gene (*tgdhfr/ts*) conferring resistance to pyrimethamine (Dessens *et al*., 1999). Transfection of EcoRI/BamHI linearized disruption cassettes, selection of transgenic lines and limiting dilution cloning were carried out as previously described (Janse *et al*., 2006b).

### Genotypic analysis of transgenic parasites

*P. berghei* genomic DNA was prepared from transfected blood stage parasite populations. White blood cells were removed by filtration over CF-11 column (Whatman) and RBCs were lysed by incubation for 20 min on ice in 0.17 M ammonium chloride. Genomic DNA was extracted using DNeasy kit (Qiagen) and subjected to diagnostic PCR and Southern blot analysis to assess successful integration. For Southern blot analysis of transgenic lines, genomic DNA was digested with *HindIII* (*Δpbgamer*) or *EcoRV* (*Δpbhado*). Blots were hybridized with a PCR-generated probe recognizing a 500–1000 bp region of the mutants. Pulse field gel electrophoresis was performed on chromosomes derived from purified blood stage parasites and the blot was hybridized against a probe recognizing the HindIII/EcoRV digest *tgdhfr/ts* fragment obtained from the pBS-TgDHFR vector.

### Exflagellation assays

Equal parts of parasite-infected blood were mixed with ookinete culture medium as described (Bushell *et al*., 2009). Following 10 min incubation at room temperature, the number of exflagellation centres and total RBCs were counted and compared with the male gametocytaemia as determined by Giemsa-stained tail blood smears.

### Macrogamete to ookinete conversion assays

Macrogamete to ookinete conversion assay was carried out as described (Billker *et al*., 2004). Briefly, ookinete culture samples (0.1 mL) from a 24 h incubated culture were harvested at 500 g for 5 min. Pellets were resuspended in 0.1 mL ookinete medium and incubated with Cy3 labelled P28 antibody (1:100 dilution) for 10 min on ice. The conversion ratio was calculated from the total number of Cy3 positive ookinetes (crescent-shaped) divided by the total number of Cy3 positive macrogametes (spherical).

### Statistics

For statistical analysis of mosquito infection, *P*-values were calculated using the Mann–Whitney *U*-test. Statistical analysis for exflagellation and ookinete conversion assays was performed using a two tailed, unpaired Student's *t*-test with error bars representing the standard error of mean.

### Imaging and enumeration of parasites

Following mosquito infection and dissection, midguts were fixed in 4% formaldehyde (v/v) (16% methanol-free, ultrapure stock diluted in PBS, Polysciences Inc.) for 20 min at room temperature and washed three times for 10 min each in PBS. Fixed midguts were mounted in Vectashield® (VectorLabs) on glass slides under sealed coverslips. Oocyst numbers were counted at 10 dpi using fluorescence microscopy under ×10 magnification. Midgut and salivary gland sporozoite numbers were calculated from homogenates of 10 *P. berghei-*infected *A. stephensi* midguts or salivary glands at days 15 or 21 dpi respectively. The numbers of sporozoites per mosquito were calculated.

### Transmission from mosquito to mouse

At least 30 *P. berghei-*infected mosquitoes were fed on anaesthetized C57BL/6 mice for 15 min at 18 and 21 dpi. Mice were allowed to recover and parasitaemia was monitored on days 5, 7, 10 and 14 following the recipient of potentially infective sporozoites by Giemsa-stained tail blood smears.

### Genetic crosses

Genetic crosses between the *Δpbgamer* or *Δpbhado* and either male-deficient or female-deficient lines were carried out as described by infecting mice with different combinations of mutant parasites (Bushell *et al*., 2009). *A. gambiae* mosquitoes were infected by feeding on these mice.

### Antibody production and detection

Purified polyclonal peptide antibodies against GAMER and HADO were obtained from the pooled sera of two immunized rabbits (Eurogentec). α-PbGAMER (IgG19) targets the COOH-terminal peptide SEKAKELLRERGYVV (EP092419; 82–96 aa) and α-PbHADO (IgG21) targets the COOH-terminal peptide VFPINFKDRNSIKNL (EP092421; 240–254 aa). For Western blot analysis, samples of purified parasites were boiled under non-reducing or reducing conditions in SDS sample loading buffer, prior to 8% or 10% (for GAMER) SDS-PAGE protein fractionation. Immuno-detection by Western blot analysis was performed according to standard procedures using α-GAMER, α-HADO and α-TAT1 antibodies at 1:250, 1:100 and 1:1000 dilutions respectively. Secondary horse radish peroxidase (HRP) conjugated goat anti-rabbit IgG and goat anti-mouse IgG antibodies were used at 1:10 000 and 1:15 000 dilutions (Promega). For IFAs, purified inactivated gametocyte and ookinete pellets were re-suspended in PBS, smeared on glass slides and allowed to air dry prior to fixation in 4% paraformaldehyde in PBS for 10 min. Fixed parasites were then permeabilized with 0.2% Triton X-100 in PBS for 5 min. Following blocking, α-GAMER and α-HADO antibodies were used at 1:100 dilution, α-P28 antibody was used at 1:500 dilution. Secondary antibodies included the ALEXA FLUOR 488 goat anti-rabbit IgG and ALEXA FLUOR 647 goat anti-mouse antibodies (Molecular Probes) all used at 1:1500 dilutions. Images were acquired using a Leica SP5 MP confocal laser-scanning microscope. Images were analysed with the ImageJ software.

### Homology modelling

Homology modelling was carried out using the homology/analogy recognition engine PHYRE2 (Kelley and Sternberg, 2009). HADO homology modelling (37% aa coverage) was generated based on the highest scoring template (PDB code IU70, 98% confidence) corresponding to the magnesium-dependent phosphatase 1. All structural figures were generated using PyMol (the PyMOL Molecular Graphics System, Version 1.3, Schrödinger LLC, Portland, USA (http://www.pymol.org/).

### Phylogenetic analysis

GAMER, HADO and their orthologous or homologous sequences were aligned using ClustalW2. BioEdit Sequence Alignment Editor was used for visualization of alignments.

## Supporting information

Fig S1 Gene expression in midgut samples used for the microarray experiments. A. *GFP* transcript levels were monitored by qRT-PCR and normalized against the average GFP expression across all the time points and are presented in a logarithmic scale. The lowest value (2 dpi) is arbitrarily set at 10. B–D. Quantitative real-time PCR analysis of *P28*, *CTRP* and *CSP* transcript levels. Values at all time points are normalized against *GFP* expression. In A–D, the mean expression values of three independent biological replicates are shown, with each time point assayed in duplicate. Error bars indicate standard error of the mean.

Fig S2 Comparison of the expression profiles of 12 genes obtained by DNA microarrays and real-time qRT-PCR. Quantitative real-time PCR data were obtained from three independent replicate infections of *A. gambiae* mosquitoes with the *P. berghei* 259c12 line, each assayed in two technical replicates. Mean fold regulation and standard errors of the mean (SEM) are shown. PlasmoDB Gene IDs are indicated.

Fig S3 Multiple sequence alignment of *Plasmodium* GAMERs. Sequence alignment of *P. berghei* GAMER (PbGAMER; PBANKA_122540) and its orthologues in *P. yoelii* (PyGAMER; PYYM_1228100); *P. chaubaudii* (PcGAMER; PCHAS_122600), *P. knowlesi* (PkGAMER; PKH_011340); *P. vivax* (PvGAMER; PVX_093500) and *P. falciparum* (PfGAMER; PF3D7_0805200). Coloured blocks outline the conserved among all species residues. Alignment was carried out using the ClustalW2; BioEdit Sequence Alignment Editor was used for visualization.

Fig S4 Multiple sequence alignment of *Plasmodium* HADOs. The putative HAD-like domain is marked by grey boundaries. This region shows high conservation among all the orthologues. The conserved residues involved in putative phosphor transfer catalysis are marked by red outlines and arrows. *P. berghei* (PbHADO; PBANKA_060390); *P. yoelii* (PyHADO; PY05386); *P. chaubadii* (PcHADO; PCHAS_060570), *P. knowlesi* (PkHADO; PKH_130370), *P. vivax* (PvHADO; PVX_084290) and *P. falciparum* (PfHADO; PF3D7_120520). Coloured blocks indicate residues conserved among all species. Alignment was carried out using the ClustalW2; BioEdit Sequence Alignment Editor was used for visualization.

Fig S5 Structural model of *Plasmodium* HADOs. 3D homology modelling of *P. berghei* HADO (PBANKA_060390) based on the magnesium-dependent phosphatase 1 (Mdp-1). The 3D model contains four conserved loops (grey) supported by a α/β (yellow and blue) core structure. The putative conserved residues aspartic (D), serine (S), arginine (R) and aspartic (D) on the conserved loops involved in the catalysis of phosphoryl transfer are shown in stick form. These residues are conserved in all the orthologues of HADO: *P. yoelii* (PY05386), *P. chaubadii* (PCHAS_0605700), *P. knowlesi* (PKH_130370), *P. vivax* (PVX_084290) and *P. falciparum* (PF3D7_120520).

Fig S6 Generation and genotypic analysis of *Δpbgamer and Δpbhado* mutant parasites. (A–C) Schematic representation of the native (wt) locus and the modified gene locus for each gene. Each disruption vector carries gene targeting sequences for *GAMER* (A) or *HADO* (B), which flank the pyrimethamine based selectable marker TgDHFR/TS. The pBS-TgDHFR/TS gene disruption vector is also presented for each gene in the middle. Integration of the ApaI/BamHI linearized vector results in replacement of the gene of interest. (C) Genotyping of *Δpbgamer* and *Δpbhado* mutant parasites by PCR-based analysis of the genomic DNA from *wt* and cloned parasites show that the *Δpbgamer* or *Δpbhado* locus is only present in each of the ko lines. (D) Southern blot analysis of genomic DNA from cloned and *wt* parasites digested with restriction enzymes. Successful integrations show a 3–4 kb band shift as a result of insertion of the TgDHFR/ts cassette into each respective locus.

Table S1 *P. berghei* differentially regulated genes in the *A. gambiae* midgut. Genes exhibiting statistically significant differential regulation across the infections data, assessed using a one-way ANOVA following correction with the Benjamini-Hochberg hypergeometric test (*P* ≤ 0.05). The data are presented as log_2_-transformed signal intensity ratios of normalized *wt*-infected midguts vs. StR (standard reference). The expression data for 1639 genes are shown. Gene PlasmoDB IDs and functional annotation as well as *P. berghei* orthologues and their annotated gene description in other *Plasmodium* species are presented. Pb, *P. berghei*; Py, *P. yoelii*; Pc, *P. chabaudi*; Pk, *P. knowlesi*; Pv, *P. vivax*; Pf, *P. falciparum*; orth; orthologues.

Table S2 Co-expression clusters of developmentally regulated *P. berghei* genes in the *A. gambiae* midguts. The table includes 564 differentially regulated *P. berghei* genes in the *A. gambiae* midgut (*P* ≤ 0.05) showing at least 0.8 log_2_-transformed fold difference between their minimum and maximum expression. They are presented according to their classification into 12 co-expression gene clusters (six main clusters, of which four are divided into sub-clusters). *P. berghei* proteomic and transcriptomic data of asexual blood stages, gametocytes, ookinetes, oocysts (day 9–12) and sporozoites (day 20–24) and orthologous *P. falciparum* proteomic and transcriptomic data from asexual blood stages, gametocytes/gametes, oocysts (days 7–8), oocyst derived sporozoites (days 13–14) and salivary gland derived sporozoites (days 18–22) are shown. AP2_*cis* elements are presented for CL3. Gene PlasmoDB IDs and functional annotation as well as *P. berghei* orthologues and their annotated gene description in other *Plasmodium* species are also presented. Pb, *P. berghei*; Py, *P. yoelii*; Pk, Pc, *P. chabaudi*; *P. knowlesi*; Pv, *P. vivax*; Pf, *P. falciparum* (1) (Hall *et al*., 2005); (2) (Khan *et al*., 2005); (3) (Raibaud *et al*., 2006); (4) (Florens *et al*., 2002); (5) (Lasonder *et al*., 2002); (6) (Lasonder *et al*., 2008); (7) (Le Roch *et al*., 2003); (8) (Young *et al*., 2005).

Table S3 Effect of *Δpbgamer* and *Δpbhado* mutants on oocyst development in *A. gambiae* and *A. stephensi* mosquitoes.

Table S4 Effect of *Δpbgamer* and *Δpbhado* mutants on parasite development in *A. stephensi* and *A. gambiae* mosquitoes.

Table S5 Primers for qRT-PCR, RT-PCR and generation of *Δpbgamer* and *Δpbhado* mutants.

Table S6 Genetic crosses between *Δpbgamer* and female or male gamete-deficient mutants.
