# Supplementary figures and images for: Characterization of Plasmodium developmental transcriptomes in Anopheles gambiae midgut reveals novel regulators of malaria transmission
Source: Cell Microbiol. 2014 Oct 31;17(2):254–68. doi: 10.1111/cmi.12363 (PMC4371638; doi:10.1111/cmi.12363)

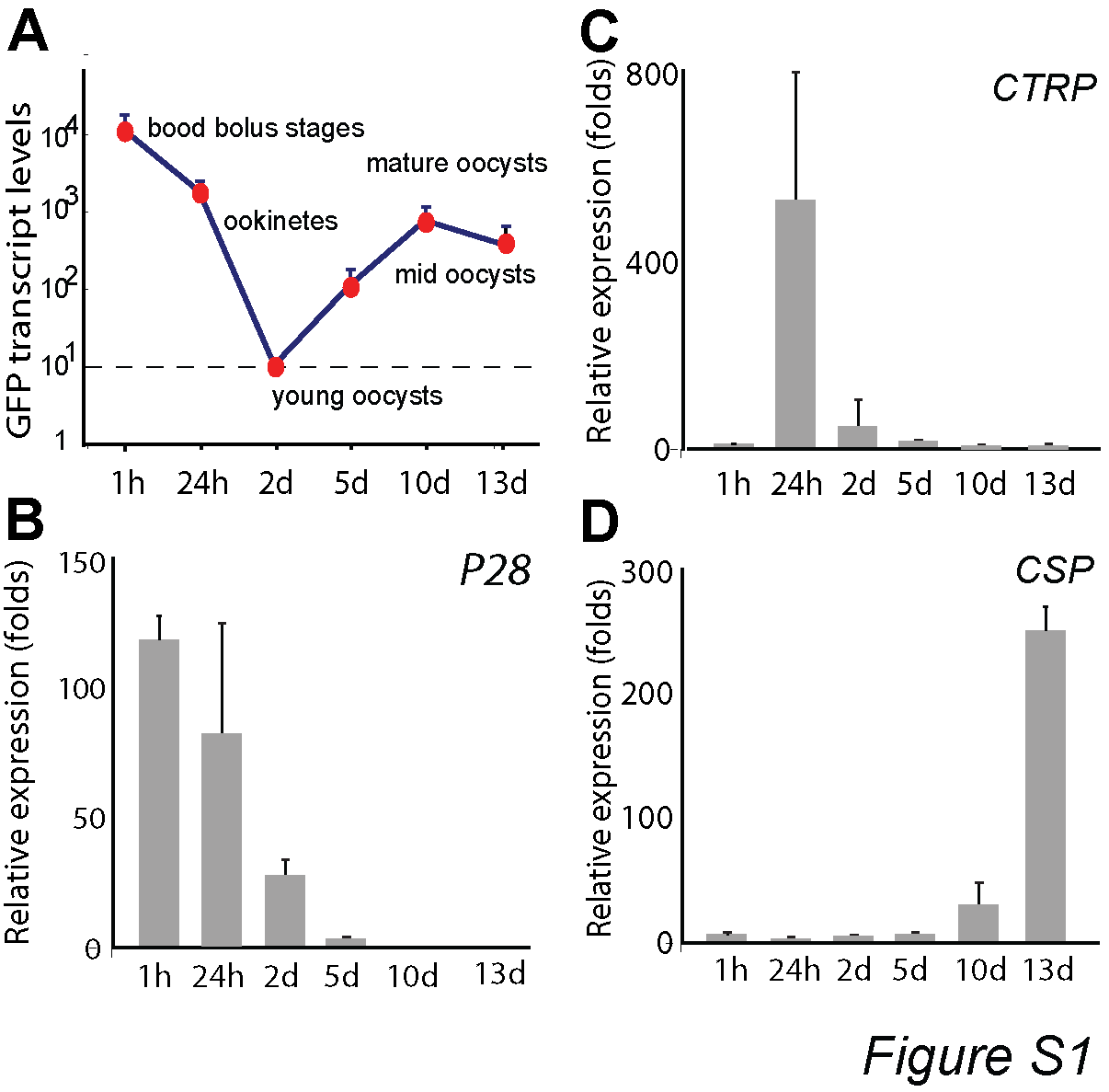

Supplement: Fig S1 — Gene expression in midgut samples used for the microarray experiments. A. GFP transcript levels were monitored by qRT-PCR and normalized against the average GFP expression across all the time points and are presented in a logarithmic scale. The lowest value (2 dpi) is arbitrarily set at 10. B–D. Quantitative real-time PCR analysis of P28, CTRP and CSP transcript levels. Values at all time points are normalized against GFP expression. In A–D, the mean expression values of three independent biological replicates are shown, with each time point assayed in duplicate. Error bars indicate standard error of the mean. [file cmi0017-0254-sd1.tif]

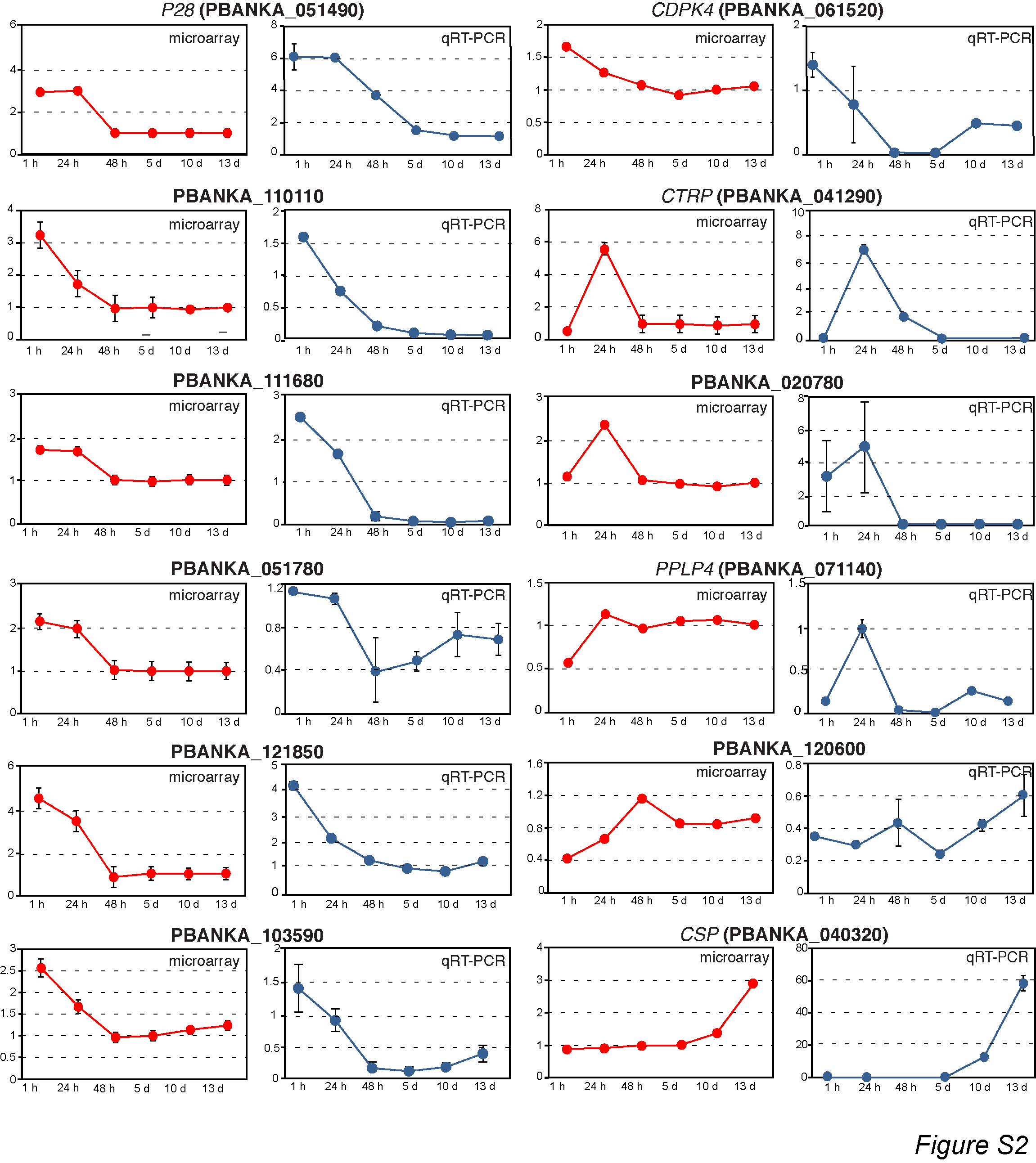

Supplement: Fig S2 — Comparison of the expression profiles of 12 genes obtained by DNA microarrays and real-time qRT-PCR. Quantitative real-time PCR data were obtained from three independent replicate infections of A. gambiae mosquitoes with the P. berghei 259c12 line, each assayed in two technical replicates. Mean fold regulation and standard errors of the mean (SEM) are shown. PlasmoDB Gene IDs are indicated. [file cmi0017-0254-sd2.tif]

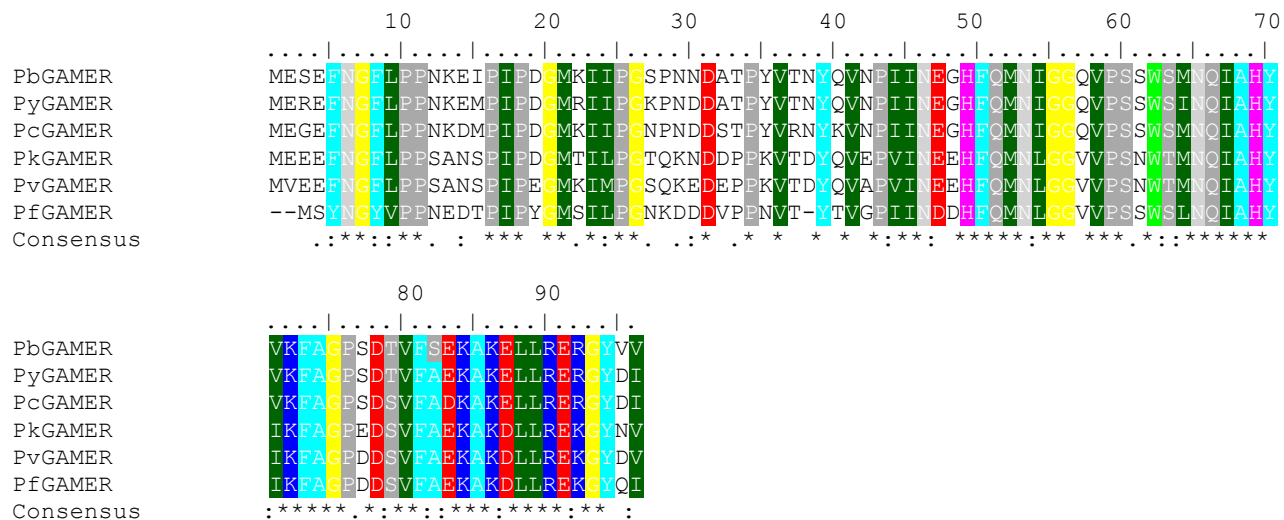

Figure S3

Supplement: Fig S3 — Multiple sequence alignment of Plasmodium GAMERs. Sequence alignment of P. berghei GAMER (PbGAMER; PBANKA_122540) and its orthologues in P. yoelii (PyGAMER; PYYM_1228100); P. chaubaudii (PcGAMER; PCHAS_122600), P. knowlesi (PkGAMER; PKH_011340); P. vivax (PvGAMER; PVX_093500) and P. falciparum (PfGAMER; PF3D7_0805200). Coloured blocks outline the conserved among all species residues. Alignment was carried out using the ClustalW2; BioEdit Sequence Alignment Editor was used for visualization. [file cmi0017-0254-sd3.pdf]

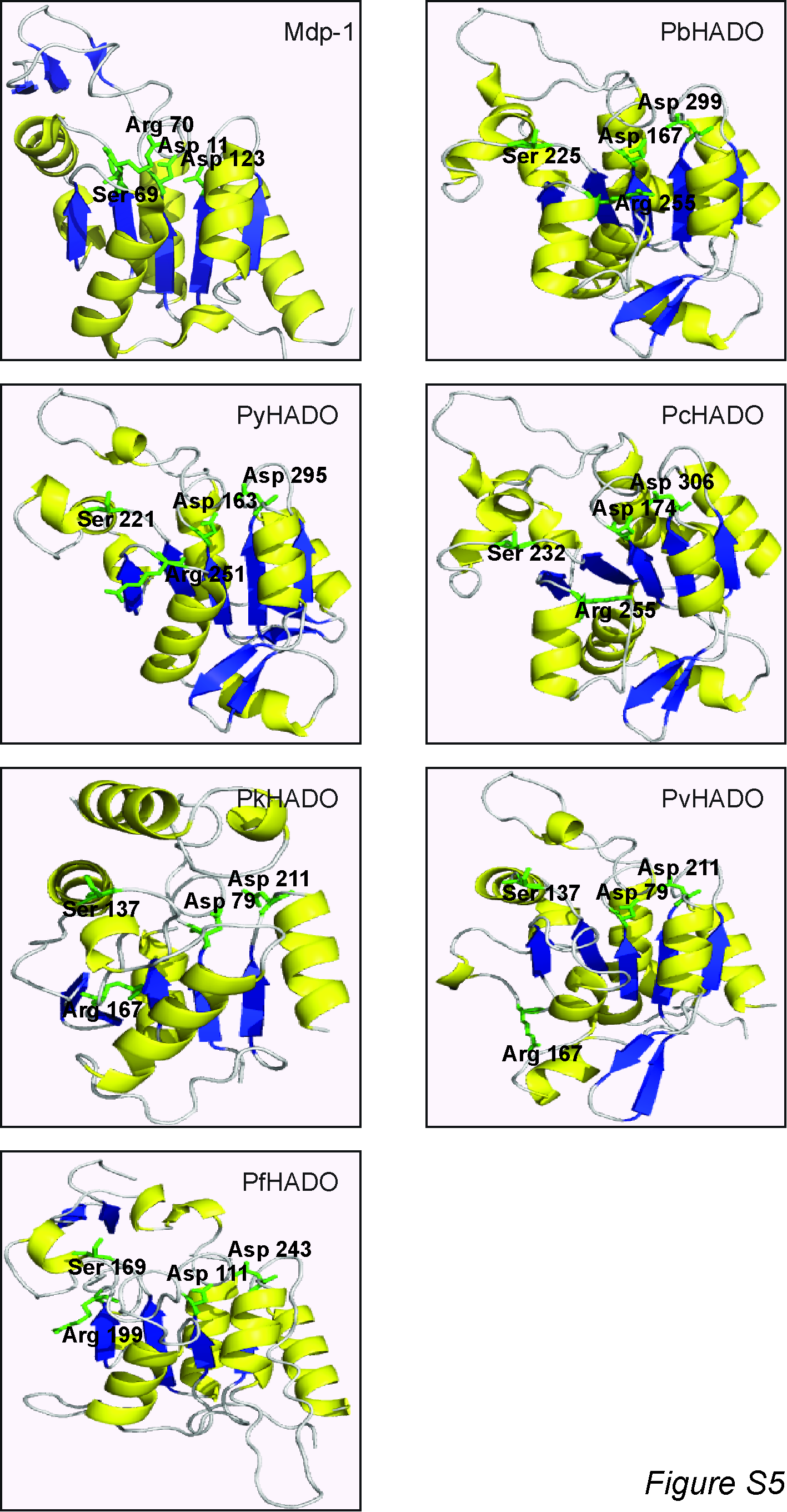

Supplement: Fig S5 — Structural model of Plasmodium HADOs. 3D homology modelling of P. berghei HADO (PBANKA_060390) based on the magnesium-dependent phosphatase 1 (Mdp-1). The 3D model contains four conserved loops (grey) supported by a α/β (yellow and blue) core structure. The putative conserved residues aspartic (D), serine (S), arginine (R) and aspartic (D) on the conserved loops involved in the catalysis of phosphoryl transfer are shown in stick form. These residues are conserved in all the orthologues of HADO: P. yoelii (PY05386), P. chaubadii (PCHAS_0605700), P. knowlesi (PKH_130370), P. vivax (PVX_084290) and P. falciparum (PF3D7_120520). [file cmi0017-0254-sd5.tif]

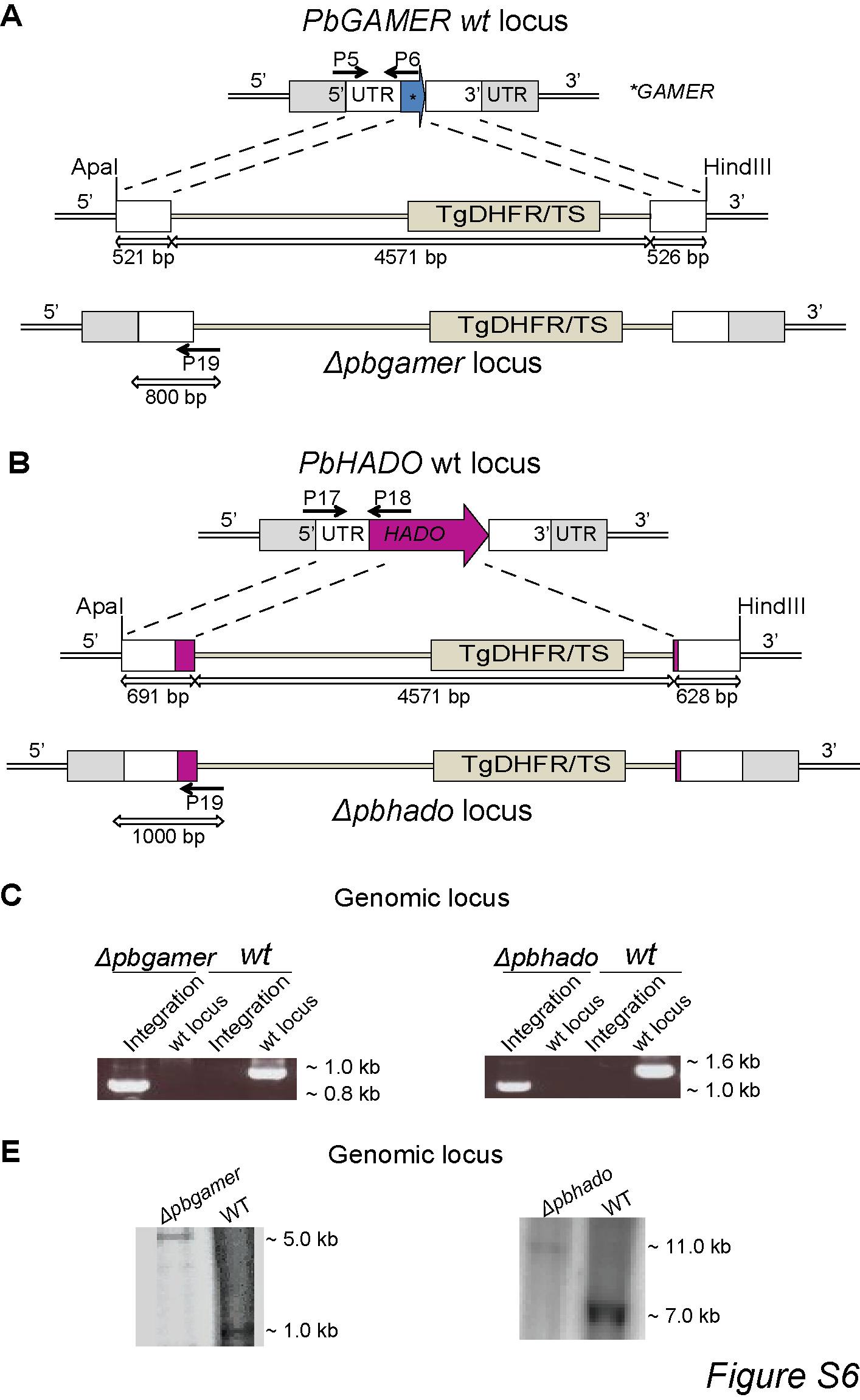

Supplement: Fig S6 — Generation and genotypic analysis of Δpbgamer and Δpbhado mutant parasites. (A–C) Schematic representation of the native (wt) locus and the modified gene locus for each gene. Each disruption vector carries gene targeting sequences for GAMER (A) or HADO (B), which flank the pyrimethamine based selectable marker TgDHFR/TS. The pBS-TgDHFR/TS gene disruption vector is also presented for each gene in the middle. Integration of the ApaI/BamHI linearized vector results in replacement of the gene of interest. (C) Genotyping of Δpbgamer and Δpbhado mutant parasites by PCR-based analysis of the genomic DNA from wt and cloned parasites show that the Δpbgamer or Δpbhado locus is only present in each of the ko lines. (D) Southern blot analysis of genomic DNA from cloned and wt parasites digested with restriction enzymes. Successful integrations show a 3–4 kb band shift as a result of insertion of the TgDHFR/ts cassette into each respective locus. [file cmi0017-0254-sd6.tif]
