# Supplementary material for: Characterization of Plasmodium developmental transcriptomes in Anopheles gambiae midgut reveals novel regulators of malaria transmission
Source: Cell Microbiol. 2014 Oct 31;17(2):254–68. doi: 10.1111/cmi.12363 (PMC4371638; doi:10.1111/cmi.12363)
Supplement: Table S3 — Effect of Δpbgamer and Δpbhado mutants on oocyst development in A. gambiae and A. stephensi mosquitoes. [file cmi0017-0254-sd9.pdf]

Table S3. Effect of *Δpbgamer* and *Δpbhado* **mutants on oocyst development in *A.gambiae* and *A.stephensi* mosquitoes.**

Time	Parasite	Number of exp	Number of midguts	Prevalence (%)	Parasite density		Parasite range	Mann-Whitney *U*-test	
					Arithmetic mean	Median		*P* value	Fold difference
***A. gambiae* infections**									
Day 10	*wt*	3	58	79%	23.47	16	0-118		
	Δpbgamer	3	60	35%	1.90	0	0-36	<0.0001	-15.2 (over 16X)
	wt	4	77	81%	33	9	0-283		
	Δpbhado	5	120	69%	14.2	3	0-175	0.0034	-2.3 (-3)
***A. stephensi* infections**									
Day 10	*wt*	2	43	100%	557	573	100-902		
	Δpbgamer	2	52	100%	48	45	1-160	<0.0001	-13.2 (-12.7)
	wt	2	40	100%	264	287	0-398		
	Δpbhado	2	41	100%	156.4	133	0-372	0.0337	-1.72 (2.1)

The table reports results from *Δpbgamer, Δpbhado* or *wt* parasite infections of *A.gambiae* and *A. stephensi. Δpbgamer, Δpbhado* or *wt* infected midguts from independent biological replicates (indicated in the third column) were pooled for each group. The total number of midguts is indicated in the fourth column. Midguts with zero parasites were also considered for calculation of the arithmetic means of parasite densities. Prevalence shows the percentage of midguts with at least one oocyst. *P* values were calculated using the Mann-Whitney *U*-test. Fold differences between *Δpbgamer, Δpbhado* and *wt* oocyst densities were computed using both the arithmetic mean and the median (in parenthesis).
