# Supplementary material for: Characterization of Plasmodium developmental transcriptomes in Anopheles gambiae midgut reveals novel regulators of malaria transmission
Source: Cell Microbiol. 2014 Oct 31;17(2):254–68. doi: 10.1111/cmi.12363 (PMC4371638; doi:10.1111/cmi.12363)
Supplement: Table S4 — Effect of Δpbgamer and Δpbhado mutants on parasite development in A. stephensi and A. gambiae mosquitoes. [file cmi0017-0254-sd10.pdf]

**Table S4**. Effect of *Δpbgamer and Δpbhado* mutants on parasite development in *A. stephensi and A. gambiae* mosquitoes.

| Parasite | Midgut sporozoites | | Salivary gland sporozoites | | Infectivity to mice | |
|---|---|---|---|---|---|---|
| | Mean | SE | Mean | SE | Day 18 | Day 21 |
| *A. stephensi* | | | | | | |
| *wt* | 71133 | 2553 | 7660 | 1973 | 2/2 | 2/2 |
| | 57612 | 3350 | 16930 | 1358 | 2/2 | 2/2 |
| | 48120 | 2052 | 7476 | 1608 | 2/2 | 2/2 |
| *Δpbgamer* | 7942 | 197 | 1100 | 35 | 2/2 | 2/2 |
| | 8685 | 364 | 4905 | 74 | 1/2 | 2/2 |
| | 7840 | 1124 | 1507 | 334 | 1/2 | 1/2 |
| *Δpbhado* | 24089 | 1076 | 3944 | 1758 | 2/2 | 2/2 |
| | 16165 | 492 | 6989 | 78 | 2/2 | 2/2 |
| | 20505 | 371 | 4418 | 1109 | 2/2 | 2/2 |
| *A. gambiae* | | | | | | |
| *wt* | 11225 | 1220 | 1695 | 74 | 1/2 | 2/2 |
| | 23216 | 1749 | 1897 | 178 | 1/2 | 2/2 |
| | 14400 | 1485 | 3656 | 1745 | 1/2 | 2/2 |
| *Δpbgamer* | 1162 | 44 | 242 | 37 | 1/2 | 2/2 |
| | 678 | 157 | 147 | 23 | 0/2 | 1/2 |
| | 1625 | 477 | 192 | 41 | 0/2 | 1/2 |
| *Δpbhado* | 5140 | 643 | 898 | 10 | 2/2 | 2/2 |
| | 7025 | 251 | 1046 | 144 | 2/2 | 2/2 |
| | 5680 | 396 | 1650 | 742 | 2/2 | 2/2 |

The table outlines quantitative sporozoite assays in *A. stephensi* and *A. gambiae* midguts and salivary glands. Three biological replicates were performed for each parasite line. In each replicate, the mean numbers of sporozoites was calculated from the number of sporozoites in suspensions from three pools of ten homogenised midguts or salivary glands, respectively, at day 21 post infection. SE shows standard error. The infectivity to mice was assessed upon blood-feeding of 30 *Δpbgamer, Δpbhado* and *wt* infected mosquitoes on two C57BL/6 mice (bite-back) at day 18 and 21 of infection, respectively. Parasitaemia in mouse blood was assessed at day 5 post-feeding and daily thereafter up to day 14 if infection was not detected.
