# Supplementary material for: Characterization of Plasmodium developmental transcriptomes in Anopheles gambiae midgut reveals novel regulators of malaria transmission
Source: Cell Microbiol. 2014 Oct 31;17(2):254–68. doi: 10.1111/cmi.12363 (PMC4371638; doi:10.1111/cmi.12363)
Supplement: Table S5 — Primers for qRT-PCR, RT-PCR and generation of Δpbgamer and Δpbhado mutants. [file cmi0017-0254-sd11.pdf]

Table S5. Primers for qRT-PCR, RT-PCR and generation of Δ*pbgamer* and Δ*pbhado* mutants.

Primer name	Sequence (5′ to 3′)	Description
PbGAMER QRT-PCR F	ATATTGGAGGCCAAGTTCCA	
PbGAMER QRT-PCR R	AGTATCGGATGGTCCAGCAA	
PbHADO QRT-PCR F	TATTCAGTAGGGGATGGGAATG	
PbHADO QRT-PCR R	TAACTTTTGACATGTCGGTTCG	
CS QRT-PCR F	GAATTCGTTAAACAGATCAGGGATAGTA	
CS QRT-PCR R	TTATACCAGAACCACATGTTACGTTACA	
P28 QRT-PCR F	AATGCACAGGTACAGGAGAAACTAAAT	
P28 QRT-PCR R	CACACTCATAATGTTTTCCAGTCAATT	
CTRP QRT-PCR F	TGCAATGATGTTTGTGGTGATTT	
CTRP QRT-PCR R	TGGTGATACATTTCTGGTTCTTATTCTT	
PBANKA_110110 QRT-PCR F	GGGGTCGACTCTATAGGCAAA	
PBANKA_110110 QRT-PCR R	GCTACAATTGCCGATTCCAT	
PBANKA_111680 QRT-PCR F	TGCTGGAATAACACCACCAG	
PBANKA_111680 QRT-PCR R	GCTGTCATCACGTTTCTTTTCA	
PBANKA_051780 QRT-PCR F	CGAGCACTGGGAGCAGTAAT	
PBANKA_051780 QRT-PCR R	TTGCAACTTCTGGAAATGGTT	
PBANKA_121850 QRT-PCR F	AGACAAGGAAAGGGGCTCAC	
PBANKA_121850 QRT-PCR R	TCCCCAATTTTTGATGTTTCA	
PBANKA_103590 QRT-PCR F	ATAGGCCATAGGGGTTCTGG	
PBANKA_103590 QRT-PCR R	GCCATGCATCTAATTCAATACC	
PBANKA_020780 QRT-PCR F	TAGAAAATGGGGAAATGATTGG	
PBANKA_020780 QRT-PCR R	AATGGCTCGTCAAATGTTTCAT	
PBANKA_120600 QRT-PCR F	TATTGGCCCGAGCTCTAAAA	
PBANKA_120600 QRT-PCR R	CACCCACCAGTCAATCCTTC	
PBANKA_061520 QRT-PCR F	TGGGGATGGTCAATTAGACAGAAAT	
PBANKA_061520 QRT-PCR R	TCGACTTCAACTTCAATAGCTGCAT	
PBANKA_071140 QRT-PCR F	TTGGGGATTTTGTATTCCATCGTTTT	
PBANKA_071140 QRT-PCR R	TCTGAACAAGCACCTTGGACATTTG	
GFP QRT-PCR F	CCTGTCCTTTTACCAGACAACCA	
GFP QRT-PCR R	GGTCTCTCTTTTCGTTGGGATCT	
PbGAMER RT-PCR F	GAAAGCGAATTCAACGGATT	
PbGAMER RT-PCR R	AGTATCGGATGGTCCAGCAA	
PbHADO RT-PCR F	CAAATGCGAGTCTAGAATGGTT	

PbHADO RT-PCR R	TGGAAATACTGGAGGTACAGACA	
P28 RT-PCR F	AATGCACAGGTACAGGAGAAACTAAAT	
P28 RT-PCR R	CACACTCATAATGTTTTCCAGTCAATT	
AMA1 RT-PCR F	TATGGGTCCAAGATATTGTAGTAATAA	
AMA1 RT-PCR R	GAATTAGCTTTACCATAAATATCTGC	
*CHT1*RT-PCR F	GCCCGCCCAGATGTAATTATA	
*CHT1*RT-PCR R	TGCCAAATTCCTACACCATCG	
PbGAMER a (P1)	TT-GGGCCC-ATTACAATTTGATATAGCCTTGCAGA	Disruption upstream target *HindIII*
PbGAMER b (P2)	CC-AAGCTT-TGTTTGAAAAATCGAAAAATAATAAGG	Disruption upstream target *HindIII*
PbGAMER c (P3)	T-GAATTC-GCAATATAAGAGTAAGGAGCCGATT	Disruption downstream target *EcoRI*
PbGAMER d (P4)	TT-GGATCC-TAAAGACGTTCTAACAGCATGAGTG	Disruption downstream target *BamHI*
PbGAMER INT F (P5)	TGCCAATCTAATGCTTTTTGGTTTAC	158 bp upstream of *PbGAMER*
PbGAMER WT R (P6)	CACAACATAACCCCTTTCGCGT	406 bp downstream of *PbGAMER*
PbHADO a (P13)	TT-GGGCCC-CTGTTGTCTTTTGTTTGCCC	Disruption upstream target *ApaI*
PbHADO b (P14)	CC-AAGCTT-TTTGGTATGGTTGCTCATTTTATTTA	Disruption upstream target *HindIII*
PbHADO c (P15)	T-GAATTC-TCCAATTAATTTCAAGGATCGAAATTC	Disruption downstream target *EcoRI*
PbHADO d (P16)	TT-GGATCC-ACTATACCATGAATTAAACCCGATTG	Disruption downstream target *BamHI*
PbHADO INT F(P17)	TCGTTATTTTATCATTTGATAATTTTACTATC	148 bp upstream of *PbHADO*
PbHADO WT R (P18)	AGGTGAATTATTTGGTCCATTCA	873 bp downstream of *PbHADO*
TgDHFR 5'UTR R (P19)	GATGTGTTATGTGATTAATTCATACAC	200 bp into the 5'UTR of *TgDHFR-TS*

Where appropriate, target restriction sites are shown as underlined italics and restriction site overhangs are also shown. The appropriate restriction enzyme is presented in the description column. F, forward; R, reverse. All primers are listed in a 5′ to 3′ direction.
