# Supplementary material for: Characterization of Plasmodium developmental transcriptomes in Anopheles gambiae midgut reveals novel regulators of malaria transmission
Source: Cell Microbiol. 2014 Oct 31;17(2):254–68. doi: 10.1111/cmi.12363 (PMC4371638; doi:10.1111/cmi.12363)
Supplement: Table S6 — Genetic crosses between Δpbgamer and female or male gamete-deficient mutants. [file cmi0017-0254-sd12.pdf]

Table S6. Genetic crosses between *Δpbgamer* and female or male gamete deficient mutants

Genetic crosses	Number of experiments	Number of midguts	Prevalence (%)	Parasite density		Parasite range	Mann-Whitney
				Arithmetic mean	Median		*P*-value
Δpbgamer	3	65	42	1.4	0	0-14	
Δpbgamer x *Δpbs47* (♂)	3	60	52	1.6	1	0-9	<0.0001
Δpbgamer x *Δpbs45/48* (♀)	3	64	50	1.6	1	0-10	<0.0001
Δpbs47 (♂)x *Δpbs45/48* (♀)	3	60	100	11.6	10	1-36	

The table reports on the outcome of *A.gambiae* infections with *P. berghei* progeny derived from crosses between *Δpbgamer* and mutants that are either female (*Δpbs47*) or male (*Δpbs48/45*) gamete deficient. The results from three independent experiments are shown. Crosses between *Δpbs47* and *Δpbs48/45* were used as a positive control. Infections were performed by directly feeding mosquitoes on mice that were co-infected with the two lines. P values were calculated using the Mann-Whitney *U*-test.
