# Supplementary material for: Characterization of Plasmodium developmental transcriptomes in Anopheles gambiae midgut reveals novel regulators of malaria transmission
Source: Cell Microbiol. 2014 Oct 31;17(2):254–68. doi: 10.1111/cmi.12363 (PMC4371638; doi:10.1111/cmi.12363)
Supplement: Fig S4 — Multiple sequence alignment of Plasmodium HADOs. The putative HAD-like domain is marked by grey boundaries. This region shows high conservation among all the orthologues. The conserved residues involved in putative phosphor transfer catalysis are marked by red outlines and arrows. P. berghei (PbHADO; PBANKA_060390); P. yoelii (PyHADO; PY05386); P. chaubadii (PcHADO; PCHAS_060570), P. knowlesi (PkHADO; PKH_130370), P. vivax (PvHADO; PVX_084290) and P. falciparum (PfHADO; PF3D7_120520). Coloured blocks indicate residues conserved among all species. Alignment was carried out using the ClustalW2; BioEdit Sequence Alignment Editor was used for visualization. [file cmi0017-0254-sd4.pdf]

```
                   10        20        30        40        50        60        70
          ....|....|....|....|....|....|....|....|....|....|....|....|....|....|

PbHADO    MKKIKSFYPFGKKSNYNIQGKETDIDELNKVQN----------KNEYDLLSYHNVNNYTNLVKINKMSNH
PyHADO    ----------------------------------------------------------------------
PcHADO    MKKIKSFYPFGKKSNYNMKTTETDVDELKKMENEKEKNISNKNKNENTLLLYRNMNNSTNLVGINNMNNH
PkHADO    MENMRNFQSGGGQINYDNYLGSLQMKGMRNAQN------------ENSHMHTAGVN--------------
PvHADO    MENMRNFQTGGDQINYDNCLGSLQINEMRNAQN------------ESSHVHTAGVN--------------
PfHADO    MDRMKCVYSLGSAINNKNYIKDMSINGMRNLEN------------DNYHNMYSNIN--------------
Consensus

                   80        90       100       110       120       130       140
          ....|....|....|....|....|....|....|....|....|....|....|....|....|....|

PbHADO    TKTIEFFRKRNDNNENVANNKTSLSSKIDKTRLEKKVDKSSEDNRISINNWNFLNNKSNKNEIYNMNNDI
PyHADO    -----------------------------------------------------------------MNNDT
PcHADO    NQILEFYKRRIDDNEKVENNRIGLNSKMDRTRLGRKVDKNSSDNRVNINNWSFLNNK---NEVYNTNNDM
PkHADO    -----------CCEEHISR------------------SSNTQIMRVENG---------------------
PvHADO    -----------CCEEHITR------------------SSNTQMMRVENG---------------------
PfHADO    -----------KHEEGILTKVQGIP--------NRAVNQNGCVIKVTNNKC----------------NNN
Consensus

                                                         HAD-like domain
                  150       160       170       180       190       200       210
          ....|....|....|....|....|....|....|....|....|....|....|....|....|....|
PbHADO    INYKLRNENKYIENKNMKENKLIKSKQYIYKKLIVFDYDDTILPTSWITVKMKLGLFDVIPKHIRQLFNK
PyHADO    INYKLRNDNKYIENKNMKENKLIKNKQYIYKKLIVFDYDDTILPTSWITVKMKLGLFDMIPKHIRQLFNK
PcHADO    TNFKLKNENKYIENKTVKEHKLIKGKQYIYKKLIVFDYDDTILPTSWITVKMRLSLYDMIPKSIRQLFNK
PkHADO    ----------------------THNKPYIYKKLIVFDYDDTILPTSWITVKMKLGLNDNIPASVRQFFFK
PvHADO    ----------------------AHNKPYIYKKLIVFDYDDTILPTSWITVKMKLGLNDNIPASVRQFFFK
PfHADO    NNNNNNNSYSYSSN-----NNLLYNKPFIYKKLIVFDYDDTILPTSWITSKMKLSLYDTIPPYINELFNK
Consensus                       .* :******************** **:*.* * **  :.::* *

                                         HAD-like domain
                  220       230       240       250       260       270       280
          ....|....|....|....|....|....|....|....|....|....|....|....|....|....|
PbHADO    LSNVVINTLRLCLTQGKLVIVTNASLEWLINSAKNYIPLVWSFIVHNNIRIVSARDTLVNTFVDSKDWKK
PyHADO    LSNVVINTLSLCLTQGKLVIVTNASLEWLINSAKNYIPLVWSFIVHNNIRIVSARDTLVNTFVDPKDWKK
PcHADO    LSIVVINTLRLCLTQGKLVIVTNASLEWLISSAKHYIPLVWSFIVHNNIRIVSARDTLFNTFVDPKDWKK
PkHADO    LSEAVIKTLSLCLTQGKLVIVTNASLEWLINSARKFIPLVWSYIILNNIRIISARDRLINSLIDPKDWKK
PvHADO    LSEVVIKTLSLCLTQGKLVIVTNASLEWLINSARKFIPLVWSYIILNNIRIISARDRLINSLIDPKDWKK
PfHADO    LSEVVINTLNLCSIQGKLVIVTNASLEWLINSAKKYIPMVWSFIMHKNIRIISARDTLINSLIDPKDWKK
Consensus  **  .**:** **  ***************.**:::**:***:*:  :****:****  *.*:::*.*****

                                         HAD-like domain
                  290       300       310       320       330       340       350
          ....|....|....|....|....|....|....|....|....|....|....|....|....|....|
PbHADO    VIFHQIINELLSPYLYNTSFICFIYSVGDGNDERNACFFISQLNQYSSCIFKSLKFLPEPTCQKLIAEHE
PyHADO    VIFHQIINELLSPYLYNTSFICFIYSVGDGNDERNACFFISQLNQYSSCIFKSLKFLPEPTCQKLIAEHE
PcHADO    VIFHQIINELLSPYLCNTSFICFIYSVGDGNDERNACFFISQLNQYSSCIFKSLKFLPEPTCQKLIAEHE
PkHADO    VIFHQIINEILSPYLCNNSVICFIYSVGDGNDERNACFFISQLNQYSSCIFKSLKFLSEPSCQKLIAEHE
PvHADO    VIFHQIINEILAPYLCNNSVICFIYSVGDGNDERNACFFISQLNQYSSCVFKSLKFLSEPSCQKLIAEHE
PfHADO    VIFHQIINEILAPYLYNTSFICFIYSVGDGNDERNACFFISQLNQYSSCIFKSLKFLSEPTCQKLIAEHE
Consensus  *********:*:*** *.*.***************************:*******.**:*********

                  360       370       380       390       400
          ....|....|....|....|....|....|....|....|....|....|
PbHADO    LFSYFMNGPN----NSPKMWSYNVSVPPVFP------------INFKDRNSIKNL
PyHADO    LFSYFMNGTN----NSPKMWNYNVPESPPFP------------INFKDRNSVKNL
PcHADO    LFSYFINGPN----NSSNVWNCNASERPVFP------------INFKDRNAIKN-
PkHADO    LFYYFFNST----CNPSAARCTSIKPIASLPRESSG--VALSTSVPKSCNSLQNV
PvHADO    LFYYFFNST----CNPSAARCTSIKPMASLPPEGSG--VVLSTSVPKSCNSLQNV
PfHADO    LFYYFFNSTNLNASNHHNNQANNIN-MNNINNNNNNNMSINSLMYKNYNSIKNV
Consensus  ** **:*..     *      .     :            *. *:::*
```

*Figure S4*
